# Prolyl 4-hydroxylase subunit alpha-2 acts as a TRIM21 ubiquitination substrate to promote papillary thyroid cancer progression via the glycolytic pathway

**DOI:** 10.1038/s41419-025-07702-0

**Published:** 2025-05-17

**Authors:** Fengping Wu, Qian Liu, Jinkang Zhang, Dongkun Xu, Xue Jiang, Kun Zhang, YaZheng Chen, Xuliang Xia, Zhiqiang Jiang, Yuhong Shi, Wenling Tu

**Affiliations:** 1 Department of Nuclear Medicine, the second Affiliated Hospital of Chengdu Medical College, China National Nuclear Corporation 416 Hospital, Chengdu, 610051 China; 2 Department of Genetics, School of Bioscience and Technology, Chengdu Medical College, Chengdu, 610500 China; 3 West China Second University Hospital, Sichuan University, Chengdu, 610041 China

**Keywords:** Cancer, Cancer metabolism

## Abstract

Papillary thyroid cancer (PTC), the predominant thyroid cancer, exhibits different adverse outcomes. However, the underlying molecular mechanisms of PTC remain inadequately elucidated. An integrative analysis of multiple mRNA-seq datasets from Gene Expression Omnibus and The Cancer Genome Atlas reveals a crucial gene, prolyl 4-hydroxylase subunit alpha-2 (P4HA2), implicated in PTC progression. This study explored the expression pattern, functional role, and underlying molecular mechanism of P4HA2 in PTC. P4HA2 expression is significantly upregulated in PTC and correlates with aggressive clinicopathological features and a poor prognosis. P4HA2 knockdown effectively suppresses PTC cell proliferation, migration, and invasion, and induces apoptosis. Conversely, P4HA2 overexpression exerts opposing effects on these cancer cell phenotypes. In vivo assessments confirmed the tumor-promoting effects of P4HA2, including subcutaneous tumor formation and multiple-organ (lung and liver) metastasis. Multi-omics analyses identified glycolytic pathway activation as a hallmark of P4HA2-driven tumorigenesis, which was further validated by measuring the extracellular acidification rate and α-ketoglutarate level. Subsequent investigation revealed that tripartite motif-containing protein 21 (TRIM21) interacts with P4HA2 through the RING domain, promoting K48-linked and K63-linked ubiquitination followed by proteasome-dependent degradation of P4HA2. The knockdown of TRIM21 significantly enhances the proliferation, migration, and invasion of PTC cells, while also inducing apoptosis. Moreover, P4H inhibitors also displayed notable anti-tumor effects in PTC cells. These findings collectively elucidate a novel mechanism through which P4HA2 potentially contributes to PTC progression, providing a promising therapeutic target.

## Introduction

According to the latest Global Cancer Observatory survey from 2020, thyroid cancer is responsible for 586,000 cancer cases globally [1]. Papillary thyroid cancer (PTC) is the most prevalent type of thyroid cancer of the endocrine system, accounting for 85–90% of thyroid cancers [2–4]. However, nearly 10–15% of patients with PTC present with aggressive tumor behavior and poor prognosis, with a stable recurrence rate of nearly 30% after 30 years post-follow-up [5]. About half of patients with PTC have lymph node metastasis, which leads to higher recurrence rate and lower survival rate [6]. Therefore, the molecular mechanism of PTC needs to be further explored and the development of more effective treatment targets for PTC are clinically significant.

Prolyl 4‑hydroxylase (P4H) is an enzyme with two identical α subunits and two β subunits, and catalyzes hydroxylation of proline to 4‑hydroxyproline, a modification particularly important in the stabilization of collagen, which is an essential component of the extracellular matrix [7]. In addition to its function in collagen stabilization, P4H can also participate in cancer development through regulation of the PI3K/AKT signaling pathway [8, 9] and endoplasmic reticulum stress, both of which are key processes in the development of cancer. The *P4HA2* gene encodes P4H α2 subunit, one of several different types of α subunits of P4H. P4HA2 is activated by the hypoxia-inducible factor 1 (HIF-1) under hypoxic conditions, causing extracellular matrix remodeling and promoting cancer metastasis [10, 11]. Studies have reported the accumulation of P4HA2 in cancer cells and its contribution in accelerating collagen deposition and metastatic dissemination in vivo, such as lung adenocarcinoma [12], hepatocellular carcinoma [13], prostate cancer [14], cervical cancer [15], glioblastoma [16] and so on. However, the specific role of P4HA2 and its mechanism of action in PTC remain largely unknown.

Metabolic reprogramming, specifically the increased uptake of glucose by cancer cells and their conversion into lactate, is a hallmark of cancer, which promotes cancer cell survival and gives these cells a growth advantage [17, 18]. Several studies have reported the importance of glycolysis in tumor development and proposed that glycolysis may be a promising therapeutic target [19, 20]. Some studies have indeed reported the role of glycolysis in PTC. For example, Huang et al. found that the demethylase FTO suppresses glycolysis and growth of PTC by decreasing the stability of APOE mRNA in an N6-methyladenosine-dependent manner [19]. Accordingly, Zhu et al. found that DNAMT3B-mediated FAM111B methylation promotes glycolysis, growth, and metastasis of PTC [21]. Further, Ji et al. found that ALKBH5-induced CircRNA NRIP1 promoted glycolysis in PTC by targeting PKM2 [22]. The above-discussed studies fully illustrate the key role of glycolysis in PTC, but whether P4HA2 acts through glycolysis is unclear; furthermore, there are currently no reports on the glycolysis of P4HA2 in PTC.

In this study, we found abnormally high expression of P4HA2 in PTC and is significantly associated with poor prognosis of patients with PTC. P4HA2 can promote the malignant biological behavior of PTC by acting as a TRIM21 ubiquitination substrate to promote PTC progression through the glycolytic pathway. More importantly, TRIM21 and P4H inhibitors could significantly inhibit the malignant biological behavior of PTC cells. These findings may potentially lead to the development of new strategies for the treatment of PTC.

## Materials and methods

### Cell culture and transfection

Human thyroid follicular epithelial cells (Nthy-ori3-1), human PTC cell lines (BCPAP, TCP-1), and human embryonic kidney cells (293 T) were cultured in Roswell Park Memorial Institute 1640 or Dulbecco’s modified Eagles medium containing 10% fetal bovine serum (FBS, VivaCell, China) and 1% penicillin-streptomycin (VivaCell, China) at 37 °C in an incubator containing 5% CO_2_. The cell lines used in this study were purchased from GuangZhou Jennio Biotech Co, Ltd (Guangzhou China), and all cells were authenticated and tested for mycoplasma contamination. The medium was replaced every 2–3 days until the cells grew to more than 90% confluence, at which point they were passaged or used for further experiments.

Plasmid constructs were prepared using different vectors. This included cloning full-length P4HA2 into pcDNA3.1-N-Flag (+) vector (Invitrogen, USA), full-length ubiquitin into pcDNA3.1-N-HA (+) vector (Invitrogen, USA), and full-length TRIM21 and TRIM21 mutant (ΔTRIM21, TRIM21 lacking the RING domain, i.e., TRIM21 lacking the cDNA encoding 16th to 54th amino acids) [23] into pCMV3-N-Myc vector (SinoBiological, China). The constructed plasmids were transfected using LipoFiter reagent (Hanbio, Cat#: HB-LF-1000). In addition, we used 2 siRNA to knock down TRIM21, and the sequence of siRNA is presented in Supplementary data 1. All transfection experiments were performed following the manufacturer’s instructions.

### Identification of key causative genes in papillary thyroid cancer through bioinformatics analysis

A total of five PTC expression profile datasets were collected, four of which were from the Gene Expression Omnibus (GEO) database (GSE3678, GSE29265, GSE35570, and GSE60542) and one from The Cancer Genome Atlas (TCGA) database. The gene expression data from both the GEO and TCGA datasets were normalized using the robust multi-array averaging (RMA) method and identified differentially expressed genes (DEGs) following the filtering criteria of |log_2_Fold Change | ≥1 and adjusted *P* value < 0.05. Survival analysis was conducted on the TCGA dataset to evaluate the relationship between the levels of gene expression and patient survival outcomes. Survival curves were prepared using the Kaplan-Meier method, and the log-rank test was employed to compare survival distributions. The expression patterns of specific genes in the thyroid gland of healthy people and PTC patients were validated by accessing the Human Protein Atlas (HPA) database (https://www.proteinatlas.org/).

### Construction of papillary thyroid cancer cells with low expression or overexpression of Prolyl 4‑hydroxylase A2 (P4HA2)

The lentivirus used in this study was purchased from Hanbio Biotechnology Incorporation (Shanghai, China). For the production of lentiviruses, first synthesize shRNAs that knock down P4HA2 or RNAs that overexpress P4HA2, and construct them onto the target vector. Then, the vector containing the target RNA was transfected into DH5 α competent cells (TIANGEN, China) and cultured for 1 h. After that, the bacterial solution containing competent cells was transferred to an agar plate for expanded culture for 14 h. Next, plasmids were extracted using the Plasma DNA purification kit (MACHEREY-NAGEL, Germany), and the extracted plasmids, virus packaging plasmids (psPAX2 vector and pMD2G vector) were transfected into 293 T cells. Subsequently, collect virus supernatant at 48 h and 72 h of transfection, and perform concentration, purification, titer detection, and sterility testing on the virus. Finally, the synthesized lentivirus was stored in a –80 °C freezer for later use.

Appropriate amounts of different lentiviruses were infected into BCPAP cells or TPC-1 cells. These lentiviruses included those that could knock down P4HA2 (HBLV-h-P4HA2-shRNA1-ZsGreen, HBLV-h-P4HA2-shRNA2-ZsGreen), lentivirus overexpressing P4HA2 (HBLV-h-P4HA2-3xflag-ZsGreen), and the corresponding control lentivirus. The information about shRNA sequences is mentioned in Supplementary Data 1. After infection, the cells were selected on puromycin for successful infection. Finally, RNA and proteins from successfully infected cells were extracted, and the knockdown or overexpression efficiency was evaluated by real-time quantitative-polymerase chain reaction (RT-qPCR) and Western blotting.

### Cell viability and colony formation assays

Cell proliferation was assessed using the Cell Counting Kit-8 kit (CCK-8; APExBIO, USA). Cells were seeded in a 96-well plate at 4 × 10^3^ cells per well and incubated for 0, 24, 48, and 72 h. Finally, the absorbance value of each well was determined at 450 nm by the microplate reader. Each group was examined in triplicate, and the relative proliferation was determined as a fold change estimated based on the absorbance of each well, and the values were normalized against that of a control group.

For the colony formation assay, 5 × 10^2^ cells were plated into 6-well plates and incubated for 2 weeks. Then, the colonies were fixed with methanol and stained using a crystal violet solution upon reaching an adequate size for visualization. Images of stained colonies were acquired and counted using ImageJ software. Each group was assessed independently in triplicate.

### Cell apoptosis assays

Cell apoptosis was detected using an annexin V-Alexa Fluor 647/PI apoptosis detection kit (YEASEN, China) following the protocol provided by the manufacturer. Briefly, the indicated cells were washed with cold phosphate buffered saline (PBS) and resuspended with staining buffer. Annexin V-Alexa (5 μL) and PI (10 μL) were added to 100 μL of the cell suspension. The mix was incubated at room temperature for 15 min and then analyzed by flow cytometry. Apoptotic cells were identified as both Annexin V + /PI− and Annexin V + /PI + .

### Cell migration and invasion assay

Cells were cultured for 24 h in serum-free medium (VivaCell, China). For the cell migration assay, cells were plated into the upper Transwell filter chamber not coated with Matrigel. For cell invasion assay, cells were seeded onto upper Transwell filter chambers coated with Matrigel (Corning, USA). Medium containing 10% FBS was added as a chemoattractant to the lower chamber to drive cell movement. Cells that migrated or invaded on the undersides of the membrane were fixed with methanol and stained with crystal violet. The images of these stained cells were acquired and counted under a microscope.

### Real-time quantitative-polymerase chain reaction

Total RNA was extracted from cells using TRIzol (MRC, USA), and then 1 μg of RNA was reverse transcribed into cDNA using the FastKing RT Kit (TIANGEN, China). Then, detection was performed using a qPCR instrument using SuperReal PreMix Plus (TIANGEN, China). The relative expression of the target gene mRNA was calculated using the 2^-ΔΔCT^ method, and GAPDH was the internal reference. The primer sequences of the target genes are mentioned in Supplementary Data 1.

### Co-immunoprecipitation and Western blotting

The protein was extracted from cells with universal protein lysis buffer (BioTeke China) and quantified using the BCA method (CWBIO, China). A 1000 μg of protein solution was prepared, to which 5 μg of antibody was added. This mixture was incubated at 4 °C with gentle rotation for 12 h. After 12 h, 30 μL of magnetic beads (MedChemExpress, USA) were added to the above mixture and incubated with rotation for 6 h at 4 °C. Then, the proteins not bound to the magnetic beads were eluted, and the remaining proteins bound to the magnetic beads were added to the loading buffer, boiled, and separated by SDS-PAGE. Finally, the proteins on the gel were transferred to a polyvinylidene fluoride membrane, blocked for 2 h, incubated with the corresponding primary and secondary antibodies, and chemiluminescence was detected. Information about the relevant antibodies is presented in Supplementary Data 1.

### Co-immunoprecipitation/liquid chromatograph mass spectrometer (Co-IP/MS)

To identify proteins interacting with P4HA2, we performed Co-IP/LC-MS and examined proteins precipitated by P4HA2 antibody (Thermo Fisher Scientific, Cat#: PA5-118136) in BCPAP cells. Briefly, following the above-mentioned immunoprecipitation (IP) steps, magnetic beads were used to enrich P4HA2 protein and the proteins interacting with it, and the Co-IP product was obtained. To the Co-IP product dithiothreitol was added and reduced at 56 °C for 30 min. Next, iodoacetamide was added and incubated at room temperature in the dark for 15 min, and then trypsin was added in a ratio of 1:50 (protease: protein) to cleave it overnight. The cleaved peptides were separated employing the EASY-nLC 1200 ultra-high performance liquid phase chromatography (UHPLC; Thermo Fisher Scientific, USA). Mobile phase A in the liquid phase system was an aqueous solution of 0.1% formic acid and 2% acetonitrile, while the mobile phase B was an aqueous solution of 0.1% formic acid and 90% acetonitrile. The peptide fragments were separated by the UHPLC and then injected into the nanospray ionization (NSI) source for ionization, and then entered into the Orbitrap Exploris 480 mass spectrometer (Thermo Fisher Scientific, USA) for analysis. The voltage for the NSI ion source was set to 2300 V, and the field asymmetric waveform ion mobility spectrometry (FAIMS) compensation voltage was set to –70 V, –45 V.

### Tissue microarray (TMA) and immunohistochemistry (IHC)

Human PTC TMA (Shanghai Outdo Biotech Company, China) consisted of 49 thyroid samples and paired samples of paracancerous tissues, and IHC was performed by the 3,3’-diaminobenzidine peroxidase method (ZSGB-BIO, China). The research related to this microarray has been approved by the Ethics Committee of the Second Affiliated Hospital of Chengdu Medical College, China National Nuclear Corporation 416 Hospital and informed consent was obtained from all subjects. Briefly, slides were blocked using the endogenous peroxidase blocker and incubated with horse serum for 1 h at room temperature. The slides were incubated overnight with anti-P4HA2 antibodies at 4 °C. Finally, the slides were incubated with horseradish peroxidase (HRP)-conjugated secondary antibodies for 30 min at room temperature, followed by microscopic observation. The Aipathwell software (Servicebio, China) was used for each field of view to evaluate and calculate the number of weak, moderate, and strong positive cells, the proportion of positive cells, positive cell density (PCD), as well as the immunohistochemical score (H-Score).

### Animal studies

All mouse studies were conducted after receiving approval from the Animal Ethics Committee of the Second Affiliated Hospital of Chengdu Medical College, China National Nuclear Corporation 416 Hospital. Male NCG nude mice (4-week-old) were procured from GemPharmatech Co., Ltd (China). The mice were randomly categorized into two groups, each comprising five animals. Stable 1 × 10^7^ TPC-1-shNC and TPC-1-shP4HA2 cells were subcutaneously injected into the experimental NCG nude mice. Following 4 weeks of the injection, the animals were euthanized, and the tumors were harvested and weighed. Furthermore, 5 × 10^5^ BCPAP-shNC and BCPAP-shP4HA2 cells were injected into the tail vein of NCG nude mice through injection. After 4 weeks, the mice were sacrificed, and tissues such as the hearts, livers, spleens, lungs, kidneys, tibiae, and fibulae were collected for fluorescence imaging of isolated small animal tissues, followed by counting of the number of tumor nodules on the lungs and livers.

After imaging, tumor and lung tissues of the above mice were collected for immunohistochemical staining and hematoxylin and eosin (H&E) staining. The steps of immunohistochemical staining were as described in Section “Tissue microarray (TMA) and immunohistochemistry (IHC)” of this article. For H&E staining, tissues were fixed with 4% paraformaldehyde overnight, embedded in paraffin, and sectioned. The slices were dewaxed and dehydrated in xylene and different concentrations of alcohol, and then stained with H&E solution for 5 min each. After staining, the different concentrations of alcohol and xylene were added to the slices for dehydration, sealed with neutral resin, and visualized and photographed under an optical microscope.

### Immunofluorescence assay

The cells were seeded into a confocal dish at a density of 1 × 10^4^ cells per well. After the cells adhered to the surface, the culture medium was aspirated and the cells were washed once with PBS. The cells were fixed with 4% paraformaldehyde for 20 min. After fixation, the cell membrane was permeabilized by adding 100–200 μL of 2% NP40 for approximately 20 min. Subsequently, the cells were blocked with 5% BSA for 2 h, and primary antibodies against P4HA2 (Thermo Fisher Scientific, Cat#: PA5-118136) and TRIM21 (Proteintech, Cat#: 67136-1-Ig) were added and incubated for 12 h. Next, secondary antibodies conjugated with fluorescent dyes specific for mouse (Proteintech, Cat#: SA00009-1) and rabbit (Invitrogen, Cat#: A10042) were then added and incubated further for 2 h. Finally, before imaging, the cells were incubated for 20 min with antifade reagent containing DAPI and photographed under a confocal microscope.

### Chemical treatments

To examine the ubiquitin-mediated degradation of P4HA2, the cells were treated with 20 μM final concentration of MG132 (MCE, China) for 8 h before they were collected, and treated with cycloheximide (CHX, MCE, China) at a final concentration of 50 μM for 0, 2, 4, 6, or 8 h before they were collected. To evaluate the potential of P4HA2 as a target for the treatment of PTC, the cells were treated with 1,4-dihydrophenonthrolin-4-one-3-carboxylic acid (1,4-DPCA, GlpBio, USA) at a final concentration of 10 μM or 30 μM for the experiments, and the cells were treated with dimethyloxalylglycine (DMOG, GlpBio, USA) at a final concentration of 150 μM for related experiments.

### Measurement of extracellular acidification rate (ECAR) and α-ketoglutarate (α-KG)

To detect the role of glycolysis pathway in PTC, we performed the ECAR assay and α-KG assay on PTC cells. Glycolysis parameters in cells were measured employing the Glycolysis Stress Test kit as per the manufacturer’s instructions (Cat#: 103020-100; Agilent). Following timed injections of 10 mM glucose, 10 μM oligomycin, or 50 mM 2-deoxyglucose, ECAR values were measured in a glycolytic stress test. The concentration of α-KG was determined in PTC cells by an α-ketoglutarate kit following the manufacturer’s instructions (Cat#: G0861W, Grace Biotechnology). Optical density was measured at 450 nm. All experiments were repeated thrice.

### mRNA sequencing and tandem mass tag (TMT) quantitative proteomics

The downstream signaling pathways affected by P4HA2 were explored by performing mRNA sequencing and tandem mass tag (TMT) quantitative proteomics on BCPAP cells with knocked-down P4HA2 and TPC-1 cells with overexpressed P4HA2. Total RNA and protein were extracted from PTC cells using TRIzol and protein lysis buffer, respectively. For RNA analysis, the mRNA sequencing library was prepared using the KC-Digital™ Stranded mRNA Library Prep Kit (Seqhealth, China), and the samples were sequenced using an Illumina NovaSeq 6000 sequencer. The raw mRNA sequencing data have been uploaded to the SRA database (https://www.ncbi.nlm.nih.gov/sra), with the SRA accession number of PRJNA1122222. Differential gene expression on the sequencing data was analyzed, and genes with |log_2_FC | ≥ 1, *P* value < 0.05 were selected as DEGs. For protein analysis, extracted proteins were quantified with BCA assay kit and digested with trypsin, and labeled with the corresponding TMT labeling reagent. MS analysis was performed using a Q Exactive HF mass spectrometer (Thermo Fisher Scientific, USA), and proteins with |log_2_FC | ≥ 0.58, *P* value < 0.05 were identified as differentially expressed proteins. Finally, the mRNA-seq and proteomics data were subjected to Gene Set Enrichment Analysis (GSEA) according to the hallmark gene set and ClusterProfiler package, and gene sets with *P* value < 0.05 were considered significantly enriched.

### Statistical analysis

Each biological experiment is repeated at least three times. Student’s *t* test or Mann–Whitney test was used to compare the two experimental groups employing GraphPad Prism software (version 9.0.0, USA). Meanwhile, One-way ANOVA test is used for comparisons of three or more groups, and Tukey method is used for inter group comparisons. The statistical data meets the condition of similar variance and numerical data were presented as the mean ± SD. *P* value < 0.05 was considered statistically significant.

## Results

### P4HA2 is highly expressed in papillary thyroid cancer (PTC) and is associated with poor prognosis of patients with PTC

**Fig. 1:**
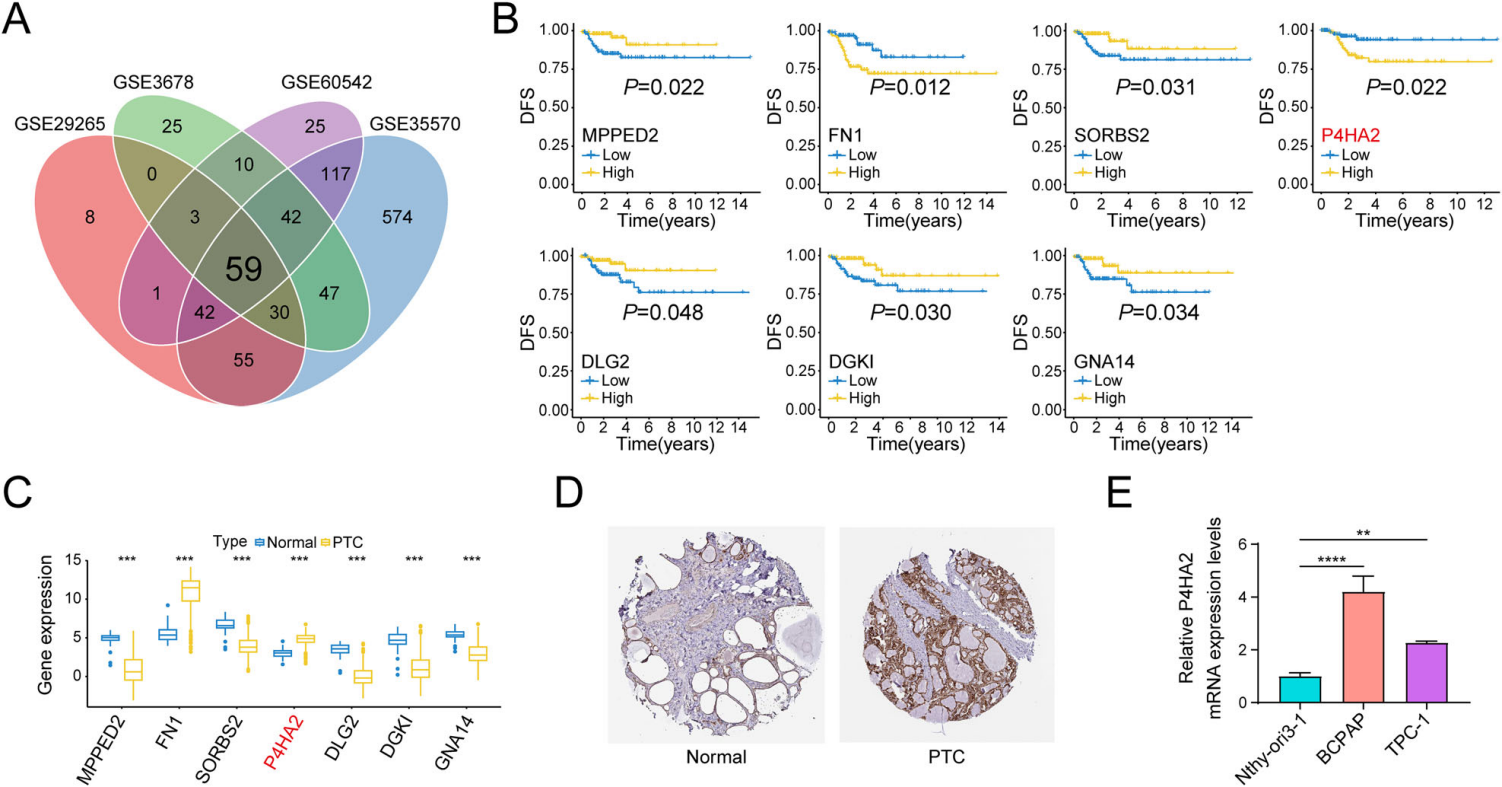
Prolyl 4-hydroxylase subunit alpha-2 (P4HA2) is expressed at high levels in papillary thyroid cancer (PTC) and is associated with poor prognosis of PTC patients. **A** Venn plot of differentially expressed genes (DEGs) for four Gene Expression Omnibus (GEO) PTC data sets. **B** Disease-free survival analysis of DEGs in PTC. **C** Boxplot of seven DEGs associated with poor prognosis in PTC. **D** Expression of P4HA2 in normal tissues and PTC tumors derived from the Human Protein Atlas (HPA) database. **E** Relative P4HA2 mRNA expression levels in healthy human thyroid cells and PTC cells. *P < 0.05; **P < 0.01; ***P < 0.001; ****P < 0.0001.

To explore potential genes that potentially influence the malignant progression of PTC, we performed a joint analysis of DEGs from the four datasets, and a total of 59 shared DEGs were obtained (Supplementary Data 2 and Fig. 1A). Subsequently, we divided PTC patients into two groups based on high and low expression of mRNA of the above 59 DEGs in the TCGA patients with PTC, and used survival analysis to determine the prognostic significance of DEGs in patients with PTC. There were statistically significant differences in disease-free survival between the high and low expression groups of seven DEGs in patients with PTC (Fig. 1B); among these, five DEGs (MPPED2, SORBS2, DLG2, DGKI, and GNA14) were expressed at low levels, while two DEGs (FN1 and P4HA2) were expressed at high levels in PTC patients (Fig. 1C). It is worth noting that the invasiveness of thyroid cancer is closely related to increased collagen deposition [24, 25]. Upon relevant literature search, we found that P4HA2 can promote the development of breast cancer and hepatocellular carcinoma by regulating collagen deposition [11, 26, 27], which may indirectly reveal the importance of P4HA2 in PTC; therefore, we selected P4HA2 for further in-depth study. Importantly, the findings from the HPA database also indicated a high expression of P4HA2 in the thyroid tissue of patients with PTC (Fig. 1D). In addition, compared with healthy human thyroid cells, the PTC cells exhibited significantly increased mRNA level of P4HA2 (Fig. 1E). In conclusion, we found that P4HA2 is highly expressed in patients with PTC and is associated with poor prognosis of patients.

### P4HA2 is highly expressed in PTC and its expression is positively correlated with tumor size

**Fig. 2:**
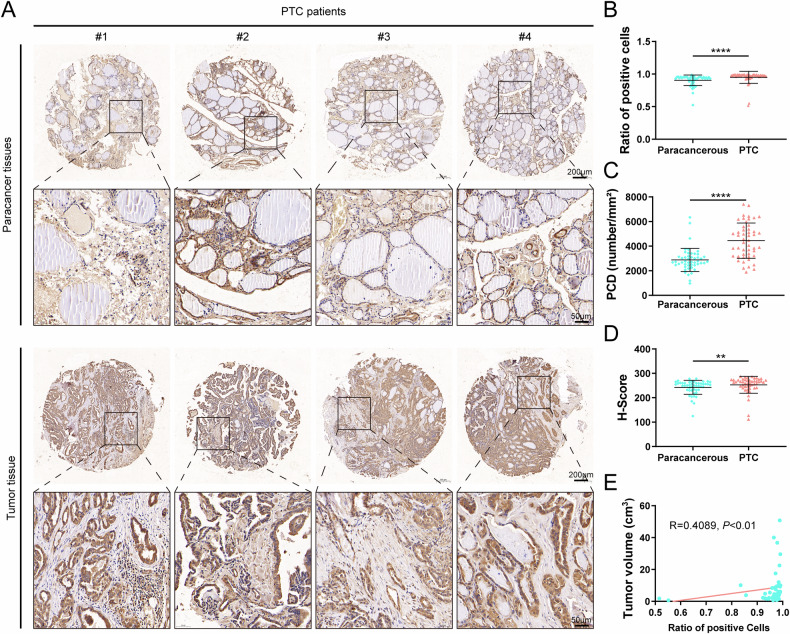
Prolyl 4-hydroxylase subunit alpha-2 (P4HA2) is highly expressed in papillary thyroid cancer (PTC). **A** Representative images of immunohistochemical staining of P4HA2 in tumors and paracancerous tissues of patients with PTC. Scatter plot of positive cell ratio (**B**), positive cell density (PCD) (**C**), and immunohistochemical score (H-Score) (**D**) of tumor tissue and adjacent tissue in patients with PTC. **E** Correlation dot plot of tumor volume and positive cell ratio in patients with PTC. Correlation test was performed by Spearman test. *P < 0.05; **P < 0.01; ***P < 0.001; ****P < 0.0001.

The role of P4HA2 in PTC was further investigated by conducting IHC staining on PTC and adjacent paracancerous tissues to determine the level of P4HA2 (Supplementary data 3). The expression of P4HA2 was significantly higher in the tumor tissues than in the adjacent paracancerous tissues (Fig. 2A and Supplementary Fig. 1), consistent with the findings from the TCGA, GEO, and HPA databases. Additionally, the number of P4HA2-positive cells was significantly higher in the tumor tissues than in the paracancerous tissue (Fig. 2B), suggesting the potential role of P4HA2 in the PTC progression. The positive cells density (PCD) and the immunohistochemical score (H-Score) for P4HA2 were significantly higher in tumor tissues than in the adjacent paracancerous tissues (Fig. 2C, D). Finally, a significant positive correlation was noted between tumor volume and the number of cells positive for P4HA2 (*R* = 0.4089, *P* < 0.01) (Fig. 2E), further corroborating the hypothesis that P4HA2 may play a role in the PTC aggressiveness. Overall, these findings indicate a very important role of P4HA2 in the progression of PTC and may be a potential therapeutic target for PTC.

### Knockdown of P4HA2 inhibits proliferation, invasiveness, migration, tumor formation and promotes apoptosis of PTC cells

**Fig. 3:**
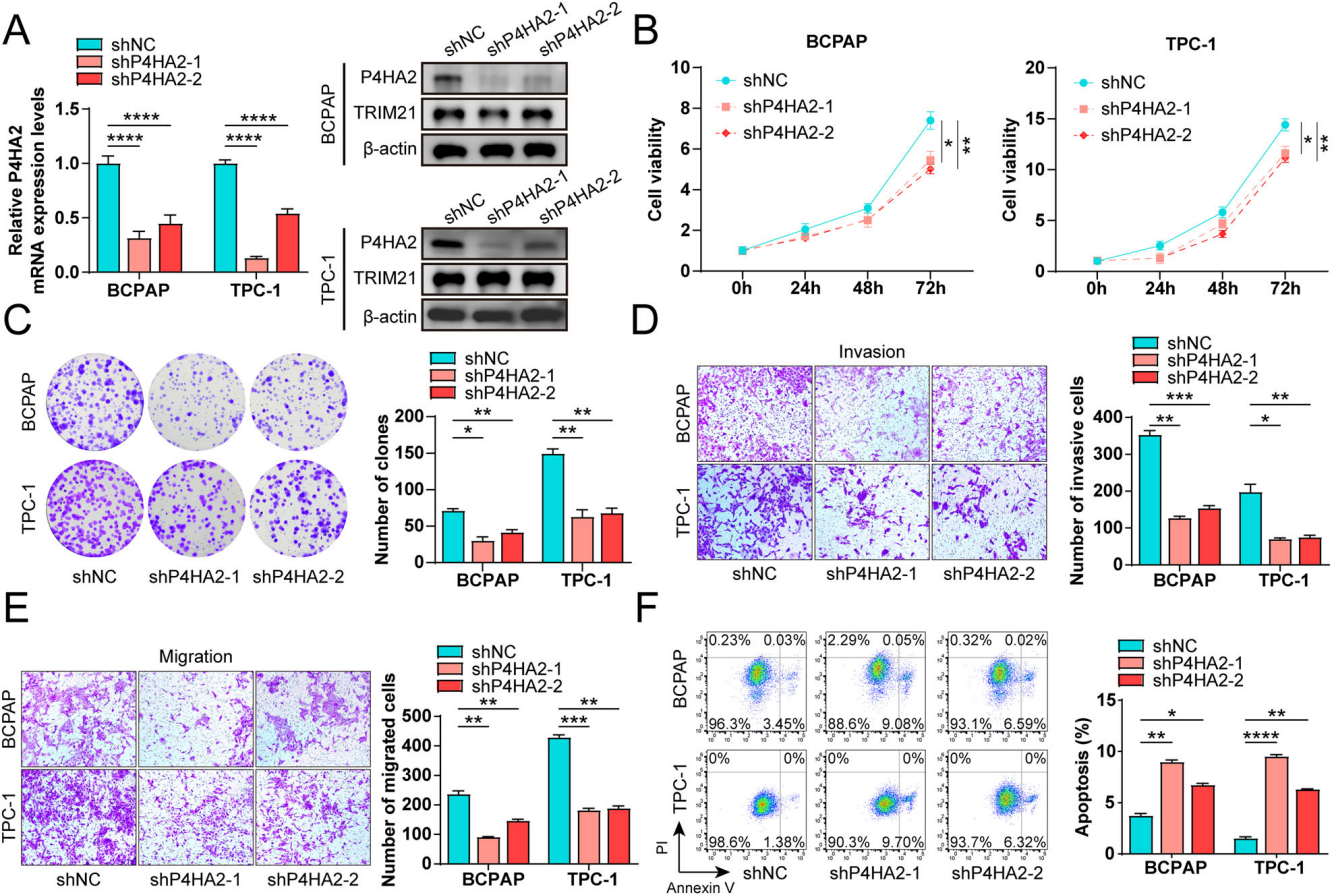
Knockdown of prolyl 4-hydroxylase subunit alpha-2 (P4HA2) inhibits proliferation, invasiveness, and migration and promotes apoptosis of papillary thyroid cancer (PTC) cells. **A** After P4HA2 knockdown in PTC cells, the expression of P4HA2 was detected by real-time quantitative-polymerase chain reaction and Western blotting assays. **B** After knocking down P4HA2 in PTC cells, cell viability was detected by CCK8 assay at 0, 24, 48, and 72 h, respectively. After P4HA2 knockdown in PTC cells, the proliferation (**C**), invasion (**D**), migration (**E**), and apoptosis (**F**) of these PTC cells were determined through colony formation assays, Transwell experiment, and flow cytometry respectively. *P < 0.05; **P < 0.01; ***P < 0.001; ****P < 0.0001.

To assess the role of P4HA2 in the malignant biology of PTC cells, we employed lentiviral-mediated gene silencing approach to downregulate the expression of P4HA2 in BCPAP and TPC-1 cells, which are poorly differentiated and normally differentiated PTC, respectively [28, 29], thus ensuring the broad representation of our study. The knockdown efficiency of P4HA2 was validated through both RT-qPCR and Western blotting assays, demonstrating a significant reduction in P4HA2 expression (Fig. 3A). Subsequently, we evaluated the impact of P4HA2 knockdown on cell proliferation by employing the CCK8 and colony formation assay. There was a marked decrease in the proliferation capacity of BCPAP as well as TPC-1 cells following P4HA2 knockdown (Fig. 3B, C). To assess the role of P4HA2 in PTC cell invasion and migration, we performed Transwell assays. These assays revealed that relative to the control group, the invasive activity of P4HA2-knockdown PTC cells was diminished, with the concomitant slowdown of the migration rate (Fig. 3D, E). Furthermore, P4HA2 knockdown significantly increased the apoptosis of PTC cells (Fig. 3F). These findings suggest that P4HA2 has a key role in the proliferation, invasion, migration and apoptosis.

### Overexpression of P4HA2 promotes proliferation, invasiveness, migration and inhibits apoptosis of PTC cells

**Fig. 4:**
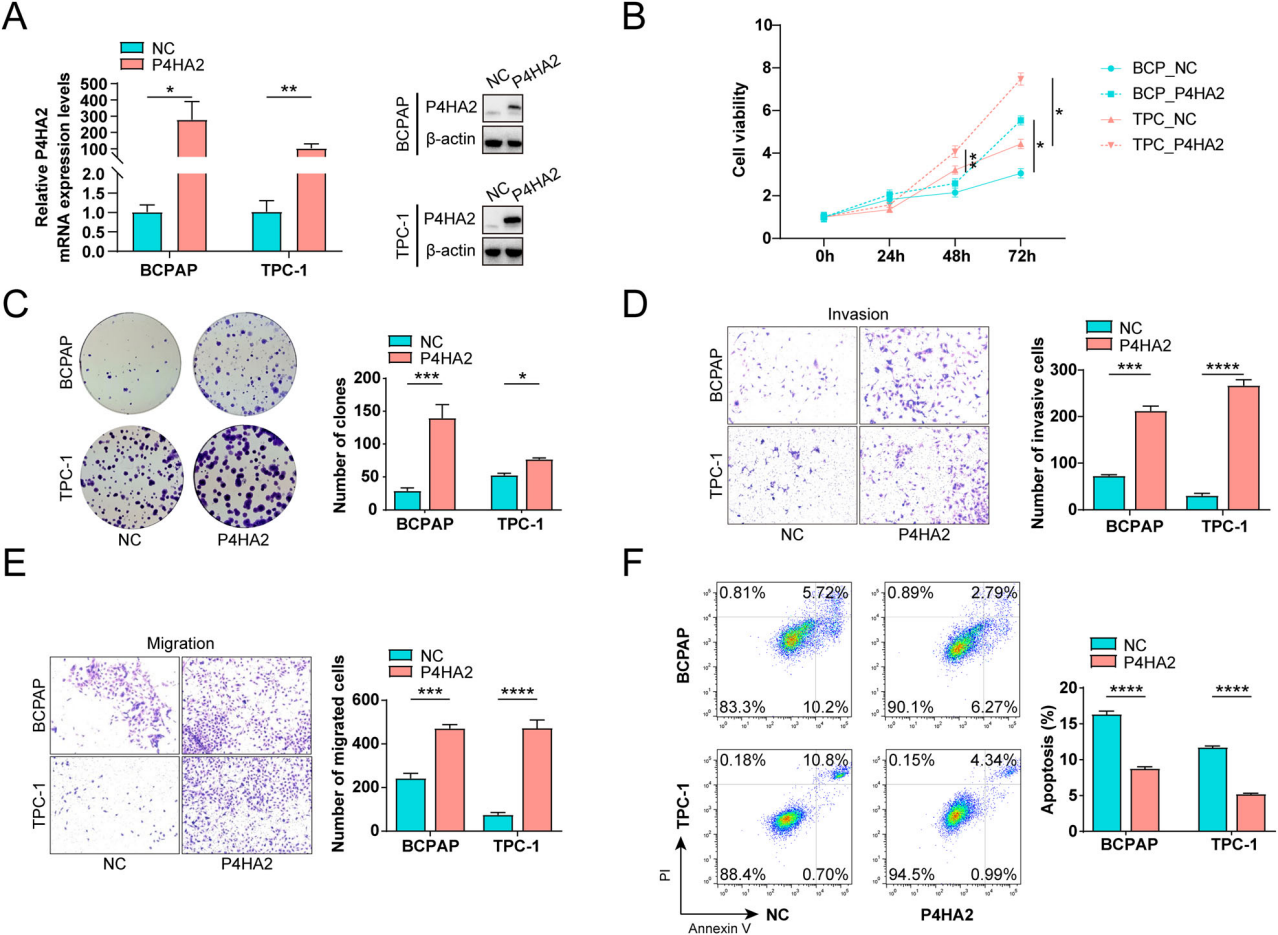
Overexpression of prolyl 4-hydroxylase subunit alpha-2 (P4HA2) promotes proliferation, invasiveness, and migration, and inhibits apoptosis of papillary thyroid cancer (PTC) cells. **A** After P4HA2 overexpression in PTC cells, real-time quantitative-polymerase chain reaction and Western blotting were performed to determine the expression of P4HA2 in PTC. **B** After overexpressing P4HA2 in PTC cells, cell viability was detected by Cell Counting Kit-8 assay at 0, 24, 48, and 72 h, respectively. After P4HA2 overexpression in PTC cells, the proliferation (**C**), invasion (**D**), migration (**E**) and the rate of apoptosis (**F**) of PTC cells were determined through colony formation experiment, Transwell experiment, and flow cytometry, respectively. *P < 0.05; **P < 0.01; ***P < 0.001; ****P < 0.0001.

To study the effect of overexpressing P4HA2 on PTC cells, we also constructed recombinant PTC cells overexpressing P4HA2 through lentivirus, and the overexpression was tested by RT-qPCR and Western blotting (Fig. 4A). In contrast to P4HA2 knockdown, the cell viability, colony formation, invasion, and migration capabilities of PTC cells were significantly enhanced after P4HA2 overexpression (Fig. 4B–E). Furthermore, the rate of PTC cell apoptosis significantly decreased after P4HA2 was overexpressed (Fig. 4F). Overall, our data suggest that P4HA2 plays a crucial role in promoting the tumor phenotype of PTC cells.

### Knockdown of P4HA2 inhibits tumor formation and multiple-organ metastasis of PTC cells

**Fig. 5:**
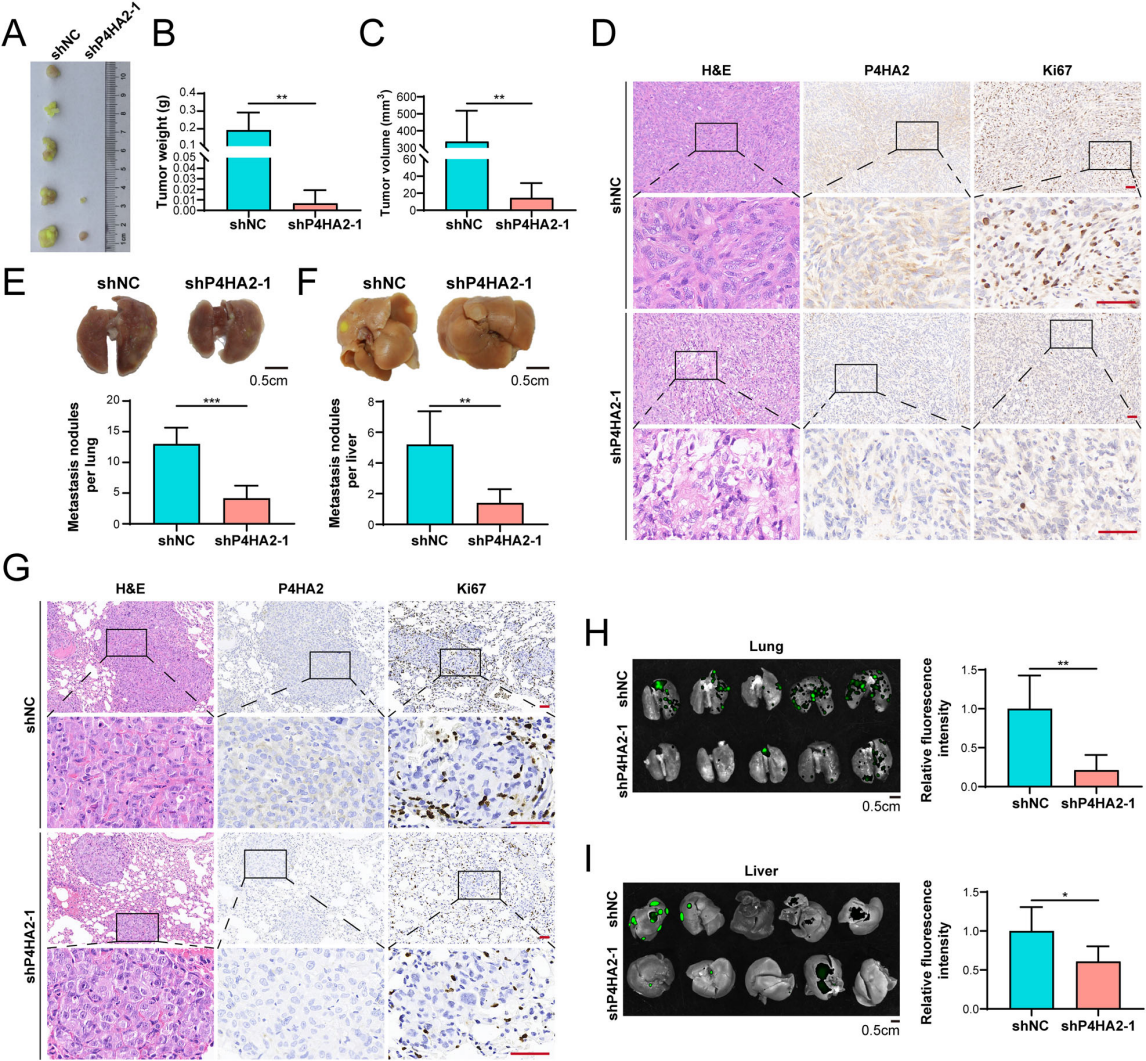
Knockdown of Prolyl 4-hydroxylase subunit alpha-2 (P4HA2) inhibited subcutaneous tumorigenesis, lung metastasis, and liver metastasis of papillary thyroid cancer (PTC) cells. Images (**A**), weight (**B**), and volume (**C**) of subcutaneous tumors in mice on day 30 after subcutaneous injection of TPC-1-shNC cells and TPC-1-shP4HA2 cells. **D** Representative images of hematoxylin and eosin (H&E) staining and immunohistochemical staining (P4HA2 and Ki67) of subcutaneous tumors in the mice. The scalebar represents 50 μm. Images of mouse lungs (**E**) and livers (**F**) on day 30th after injection of BCPAP-shNC cells and BCPAP-shP4HA2 cells in the mice tail vein. **G** Representative images of H&E staining and immunohistochemical staining (P4HA2 and Ki67) of metastatic tumors in the lungs of experimental mice. The scalebar represents 50 μm. Fluorescent images of mouse lungs (**H**) and livers (**I**) after injection of BCPAP-shNC cells and BCPAP-shP4HA2 cells in the tail vein. The green spots in the figure indicate BCPAP-shNC cells or BCPAP-shP4HA2 cells expressing green fluorescent protein (GFP). *P < 0.05; **P < 0.01; ***P < 0.001.

To further assess the in vivo effect of P4HA2 on tumor growth, we implanted P4HA2-knockdown TPC-1 cells into mice and compared their tumor growth with control mice. The tumor formation rate of P4HA2-knockdown TPC-1 cells was significantly lower, at 40% (Fig. 5A). Furthermore, the tumor growth rate in the shP4HA2 group was significantly slower relative to that in the control group, and the tumor volume and weight were significantly reduced (Fig. 5B, C). IHC analyses results showed that the knockdown of P4HA2 reduced the expression of the proliferation marker Ki67 in subcutaneous tumors (Fig. 5D). Further, we injected BCPAP-shNC cells and BCPAP-shP4HA2 cells into two groups of mice and detected the effect of P4HA2 on the metastasis of PTC cells. The metastasis of PTC cells mainly occurred in the lungs and livers (Fig. 5E, F, H, I), but there was no obvious colonization in other organs such as the heart, spleens, kidneys, and bones (Supplementary Fig. 2A). Importantly, mice injected with BCPAP-shP4HA2 cells had fewer tumor nodules in the lungs and livers compared with the mice in the control group (Fig. 5E, F, H, I). IHC results showed that P4HA2 knockdown also reduced the expression of Ki67 in lung metastatic tumors (Fig. 5G). These results cumulatively suggest that P4HA2 is important for in vitro tumorigenesis in PTC.

### Multi-omics analysis reveals a potential influence of P4HA2 on the function of glycolysis signaling pathway

**Fig. 6:**
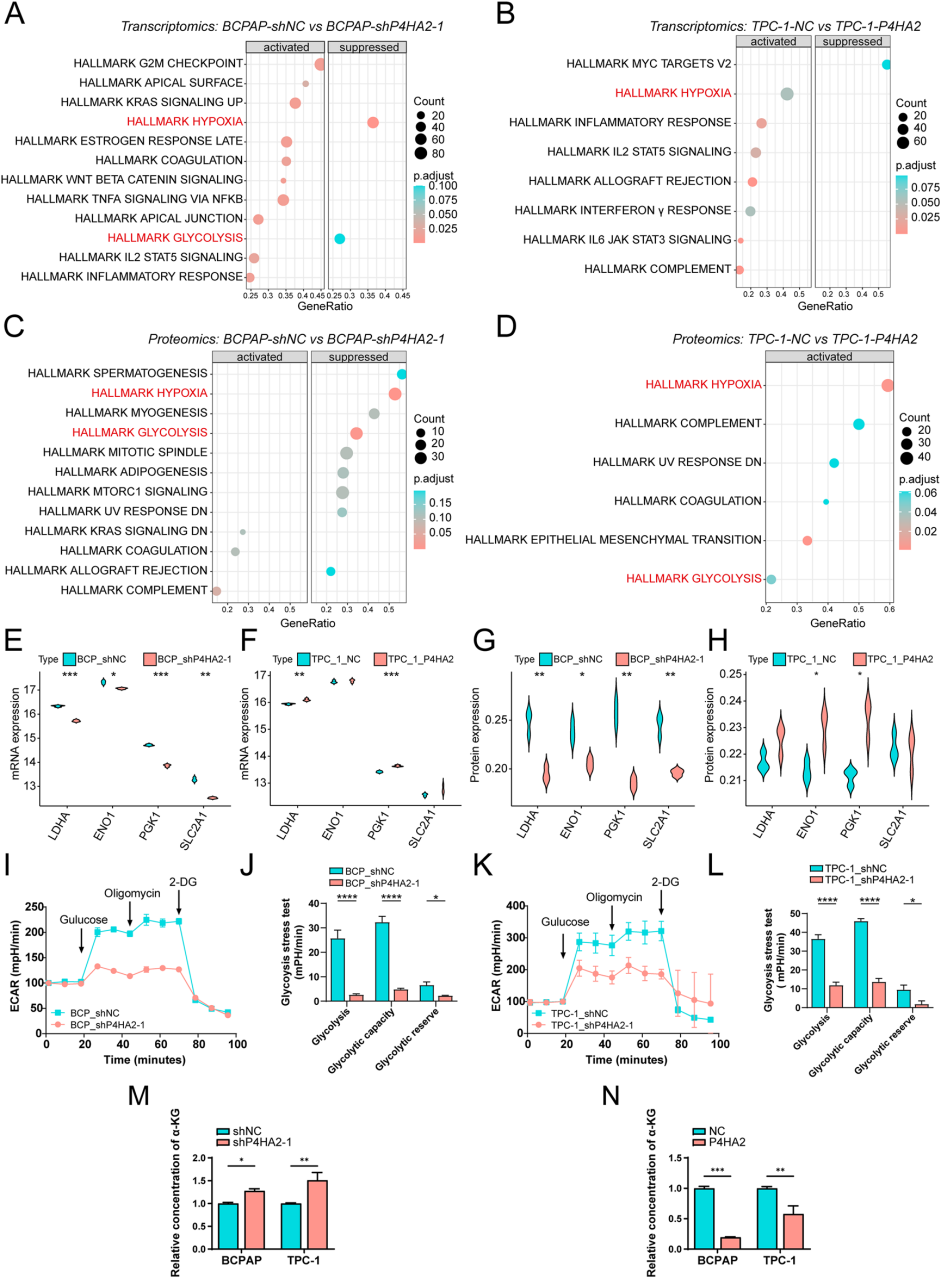
Prolyl 4-hydroxylase subunit alpha-2 (P4HA2) functions in papillary thyroid cancer (PTC) through the glycolytic pathway. After P4HA2 knockdown in BCPAP cells (**A**) or P4HA2 overexpression in TPC-1 cells (**B**), the RNA of the cells was extracted for mRNA sequencing, and gene set enrichment analysis (GSEA) was performed on the sequencing data. After P4HA2 knockdown in BCPAP cells (**C**) or P4HA2 overexpression in P4HA2 in TPC-1 cells (**D**), the proteins of the cells were extracted and analyzed by mass spectrometry (MS), and then the obtained data were subjected to GSEA. The mRNA levels of key glycolysis genes, including LDHA, ENO1, PKG1, and SLC2A1 was determined in BCPAP cells with P4HA2 knockdown (**E**) or P4HA2 overexpression in TPC-1 cells (**F**). **G**, **H** The levels of proteins expressed by these key glycolysis genes, including LDHA, ENO1, PKG1, and SLC2A1 in BCPAP cells with knocked-down P4HA2 (**E**) or TPC-1 cells overexpressing P4HA2 (**F**). **I**, **J** The glycolytic activity was measured through the extracellular acidification rate (ECAR) assay in BCPAP cells with P4HA2 knockdown. **K**, **L** Glycolytic activity was measured using the ECAR assay in TPC-1 cells with P4HA2 knockdown. The level of α-KG was measured using the α-ketoglutarate kit in PTC cells with P4HA2 knockdown (**M**) and P4HA2 overexpression (**N**). *P < 0.05; **P < 0.01; ***P < 0.001; ****P < 0.0001.

To explore the signaling pathways affected by P4HA2 in PTC, we performed transcriptomic and proteomic analyses in BCPAP cells with P4HA2 knockdown (BCPAP-shP4HA2) and control cells (BCPAP-shNC) and TCP-1 cells overexpressing P4HA2 (TCP-1-P4HA2) and control cells (TCP-1-NC) (Supplementary data 4). The results of GSEA showed that the *hypoxia* as well as *glycolysis* pathways were enriched at the transcription (Fig. 6A, B) and protein levels (Fig. 6C, D). We first tested the effect of P4HA2 knockdown or overexpression on HIF-1α in PTC cells through Western blotting, but did not observe any expected trend (Supplementary Fig. 2B, C). Furthermore, the co-IP experiments also did not reveal any interaction between P4HA2 and HIF-1α (Supplementary Fig. 2D). Analysis of the mRNA and protein expression of key genes (LDHA, ENO1, PGK1 and SLC2A1) involved in glycolysis in the transcriptomics and proteomics data revealed that surprisingly, the mRNA and protein levels of these four genes were down-regulated in BCPAP cells with P4HA2 knockdown (Fig. 6E, G), while the levels of PGK1 transcript and protein were up-regulated in TCP-1 cells overexpressing P4HA2 (Fig. 6F, H), indicating the potential effect of P4HA2 on the glycolysis pathway where the above four genes are located. To further test our hypothesis, we performed the Seahorse assay to measure ECAR to determine whether P4HA2 knockdown could affect glycolytic metabolism in PTC cells. The results showed reduced ECAR in both cells after P4HA2 knockdown in BCPAP and TPC-1 cells, indicating a decline in lactate production during glycolysis (Fig. 6I, K). Furthermore, glycolysis level, glycolytic capacity, and glycolytic reserve also decreased significantly (Fig. 6J, L). Multiple studies have shown that α-ketoglutarate (α-KG), a TCA cycle intermediate, inhibits glycolysis and shifts the cellular metabolism toward oxidative phosphorylation [30–32]. Therefore, we tested the effect of P4HA2 on α-KG in PTCs and found that after P4HA2 knockdown, the levels of α-KG were significantly up-regulated in BCPAP cells and TPC-1 cells, while overexpression of P4HA2 had the opposite effect (Fig. 6M, N). Combined with the above-mentioned sufficient research evidence, we speculate that P4HA2 possibly promotes glycolysis in PTC by reducing the level of α-KG. Taken together, these findings suggest that P4HA2 has a crucial role in the glycolysis pathway in PTC, potentially contributing to the progression of the disease.

### P4HA2 as a ubiquitination substrate of TRIM21 promotes the progression of PTC cells

**Fig. 7:**
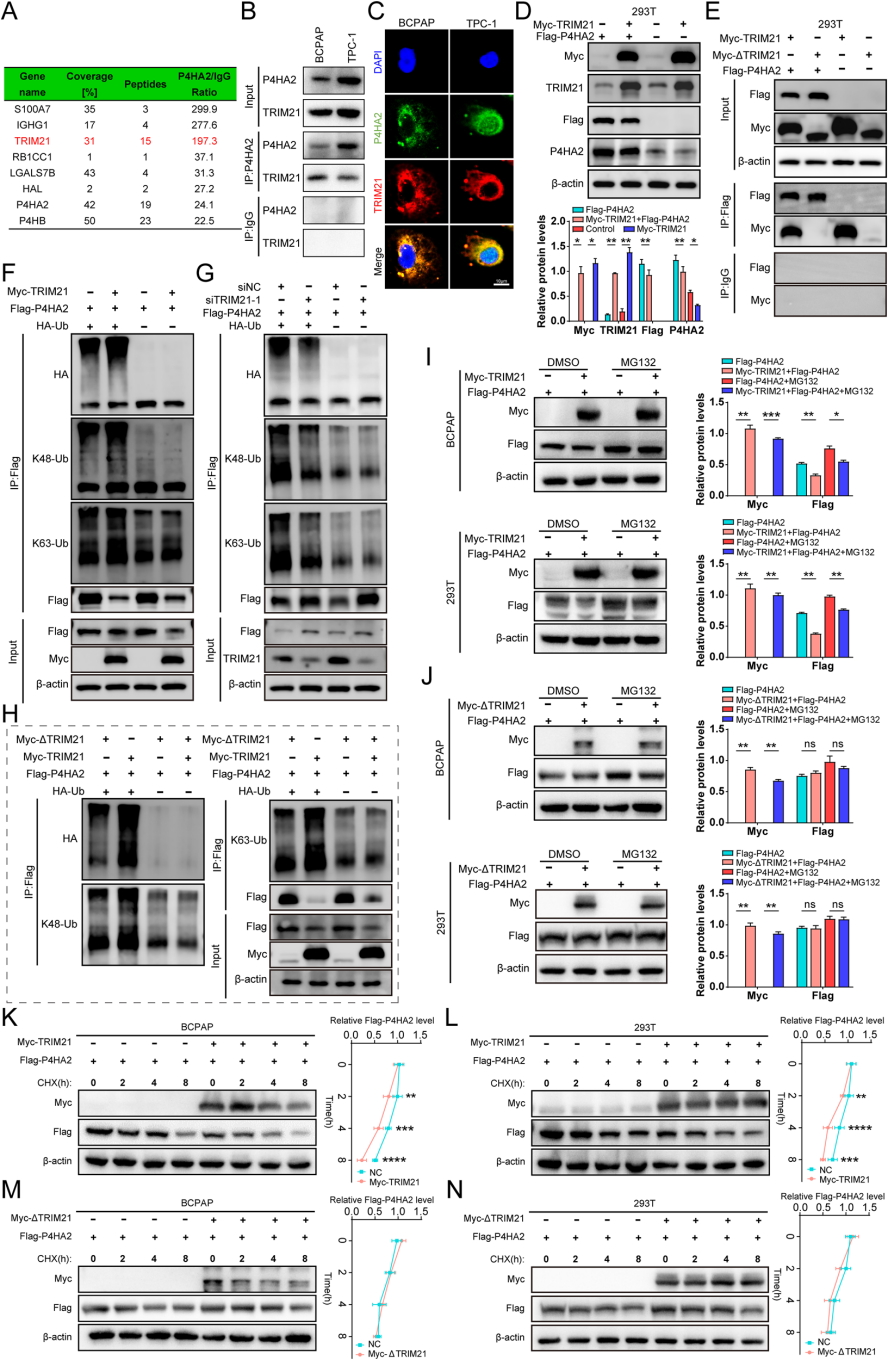
TRIM21 increases prolyl 4-hydroxylase subunit alpha-2 (P4HA2) ubiquitination through the RING domain to reduce the protein stability of P4HA2. **A** Protein from BCPAP cells, anti-P4HA2 and IgG antibodies were used for immunoprecipitation (IP), and the IP products were identified by MS. The interaction between P4HA2 and TRIM21 was verified through Co-IP (**B**) and immunofluorescence techniques (**C**). **D** After transfection in 293 T cells with the corresponding plasmid, the effect of TRIM21 overexpression on P4HA2 protein was detected by Western blotting assay. **E** The interaction between P4HA2 and ΔTRIM21 was detected by Co-IP after transfection of the corresponding plasmid. The effects of TRIM21 (**F**), siTRIM21 (**G**), and ΔTRIM21 (**H**) on P4HA2 ubiquitination were detected by Co-IP after transfection with the corresponding plasmid. **I**, **J** BCPAP and 293 T cells were transfected with corresponding plasmids for 48 h, then treated with MG132 or DMSO for 8 h, and the proteins were extracted for Western blotting. **K**–**N** BCPAP and 293 T cells were transfected with the corresponding plasmids for 48 h, and then CHX or DMSO intervention was performed for 0, 2, 4, and 8 h, and the proteins were extracted for Western blotting. *P < 0.05; **P < 0.01; ***P < 0.001; ****P < 0.0001.

In order to identify which molecules regulate P4HA2, we found many molecules that interact with P4HA2 by IP and MS analysis (Fig. 7A). Among the top five interacting molecules identified above, TRIM21, an E3 ubiquitin ligase that mediates the ubiquitination of molecules, attracted our interest. Their interactions were further confirmed by co-IP (Fig. 7B) and immunofluorescence experiments (Fig. 7C). However, P4HA2 knockdown or overexpression had no effect on the TRIM21 protein level (Fig. 3A and Supplementary Fig. 2B, C). We further found that TRIM21 overexpression could reduce the protein level of P4HA2 (Fig. 7D). Analysis of the mRNA sequencing data of PTC patients from TCGA revealed that the level of mRNA of TRIM21 did not correlate with that of P4HA2 (Supplementary Fig. 2E) and we found that overexpression of TRIM21 had no effect on the expression of P4HA2 mRNA (Supplementary Fig. 2K, L), so we speculated that TRIM21 affected P4HA2 expression at the protein level. TRIM21 protein has four structural domains, including RING, BBOX, Coiled-coil, and PRY-SPRY (Supplementary Fig. 2F). Several studies have shown that TRIM21 mainly functions as a ubiquitinase through the RING domain [33–35]. Therefore, we hypothesized that TRIM21 interacts with P4HA2 through the RING domain, and constructed a plasmid expressing TRIM21 mutant with a deleted RING domain (Myc-ΔTRIM21) (Supplementary Fig. 2F). The co-IP confirmed that when the RING domain of the TRIM21 protein was deleted, the interaction between TRIM21 and P4HA2 disappeared (Fig. 7E), indicating that TRIM21-mediated the ubiquitination of P4HA2 involves the RING domain of TRIM21. Related studies have reported that TRIM21 or other ubiquitin-related enzymes mediates the degradation of various substrate proteins through K48-linked and K63-linked ubiquitination [36, 37]. Therefore, we used Myc-TRIM21 (or Myc-ΔTRIM21), Flag-P4HA2, HA-Ubiquitin (HA-Ub) and siTRIM21 to co-transfect 293 T cells for ubiquitination assay. TRIM21 increased the ubiquitination level of P4HA2 (Fig. 7F), but ΔTRIM21, lacking the RING domain, could not cause this effect, demonstrating that TRIM21 mediates P4HA2 ubiquitination through the RING domain (Fig. 7H). In addition, knockdown of TRIM21 reduces the ubiquitination level of P4HA2 (Fig. 7G). K48 and K63 is the major ubiquitin linkage type that causes the ubiquitination of P4HA2 by TRIM21 (Fig. 7F–H). Next, to determine whether the ubiquitination degradation of P4HA2 caused by TRIM21 is mediated through a ubiquitin-proteasome-dependent pathway, we treated BCPAP and 293 T cells with MG132 (a proteasome inhibitor). Following treatment, we found that the changes in endogenous and exogenous P4HA2 protein levels caused by TRIM21 but not ΔTRIM21 was diminished, indicating that TRIM21 mediates the degradation of P4HA2 through the ubiquitination pathway (Fig. 7I, J and Supplementary Fig. 2G–J). Furthermore, we explored the effect of TRIM21 on P4HA2 protein stability by performing CHX assay (CHX is an inhibitor of translation). Under the treatment of CHX, the half-life of P4HA2 protein in BCPAP cells and 293 T cells transfected with Myc-TRIM21 was reduced (Fig. 7K, L), while no such effect was observed in cells transfected with Myc-ΔTRIM21 (Fig. 7M, N), indicating that TRIM21 decreased the stability of P4HA2 protein. Taken together, these data demonstrate that TRIM21 reduces the stability of P4HA2 protein through the RING domain and the K48-linked and K63-linked ubiquitin-proteasome pathway.

### Knockdown of TRIM21 promotes proliferation, invasiveness, migration and inhibits apoptosis of PTC cells

**Fig. 8:**
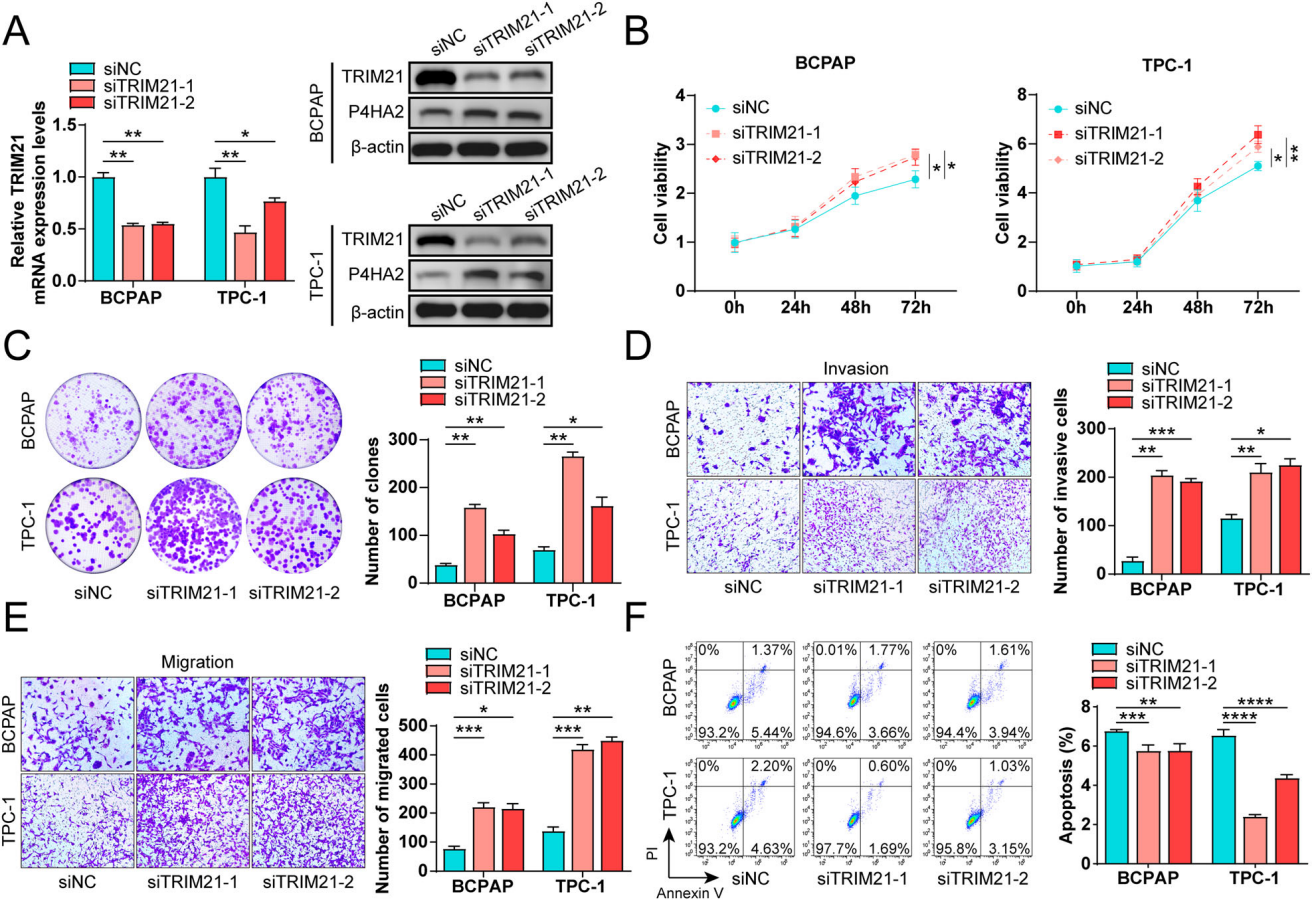
Knockdown of tripartite motif-containing protein 21 (TRIM21) promotes proliferation, invasiveness, and migration and inhibits apoptosis of papillary thyroid cancer (PTC) cells. **A** After TRIM21 knockdown in PTC cells, the expression of TRIM21 was detected by RT-qPCR and Western blotting assays. **B** After TRIM21 knockdown in PTC cells, cell viability was detected by CCK8 assay at 0, 24, 48, and 72 h, respectively. After TRIM21 knockdown in PTC cells, the proliferation (**C**), invasion (**D**), migration (**E**), and apoptosis (**F**) of these PTC cells were determined through colony formation assays, Transwell experiment, and flow cytometry respectively. *P < 0.05; **P < 0.01; ***P < 0.001; ****P < 0.0001.

To directly investigate the function of TRIM21 in the malignant phenotype of PTC cells, we generated PTC cells with TRIM21 knockdown using small interfering RNA (siRNA). The knockdown efficiency was validated by RT-qPCR and Western blotting analyses (Fig. 8A). Post-knockdown, we observed a significant enhancement in cell viability, colony formation, invasion, and migration capabilities of PTC cells (Fig. 8B–E). Furthermore, TRIM21 knockdown led to a marked reduction in the apoptosis rate of PTC cells (Fig. 8F). Collectively, these findings suggest that TRIM21 plays a crucial role in suppressing the tumorigenic properties of PTC cells.

### P4H inhibitors inhibit proliferation, invasiveness, migration and promote apoptosis of PTC cells

**Fig. 9:**
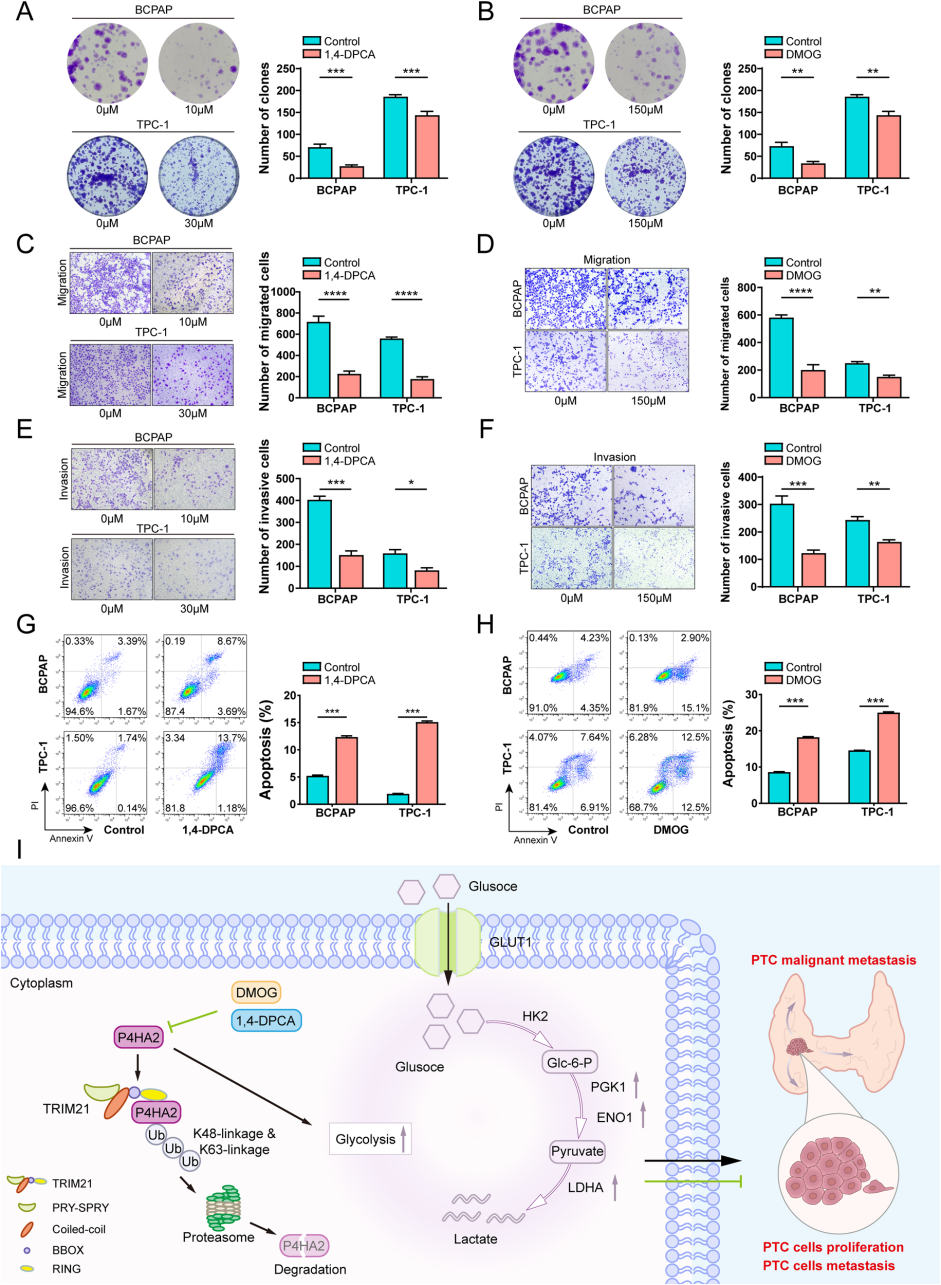
Prolyl 4-hydroxylase (P4H) inhibitors stall proliferation, migration, and invasion and promotes apoptosis of PTC cells. **A** After treating BCPAP cells with 10 μM and TPC-1 cells with 30 μM 1,4-dihydrophenonthrolin-4-one-3-carboxylic acid (1,4-DPCA), respectively, the cell proliferation was detected by colony formation assay. **B** After treating BCPAP and TPC-1 cells with 150 μM dimethyloxalylglycine (DMOG), the cell proliferation was detected by colony formation assay. The BCPAP cells were treated with 10 μM 1,4-DPCA and TPC-1 cells were treated with 30 μM 1,4-DPCA, and the cell migration (**C**), invasion (**E**) and apoptosis (**G**) were detected by Transwell assay and flow cytometry. The BCPAP and TPC-1 cells were treated with 150 μM DMOG for 48 h, and cell migration (**D**), invasion (**F**) and apoptosis (**H**) were detected by Transwell assay and flow cytometry. **I** The mechanistic diagram of this study is presented here. On the one hand, P4HA2, as a substrate of TRIM21, undergoes K48 and K63 linked ubiquitination, leading to its degradation. On the other hand, P4HA2 can cause malignant changes in PTC cells by promoting the expression of key enzymes of the glycolysis pathway, such as PGK1, ENO1, and LDHA, while the use of P4H inhibitors can reverse this change. *P < 0.05; **P < 0.01; ***P < 0.001; ****P < 0.0001.

To further explore the strategy of targeting P4HA2 to treat PTC, we investigated the potential therapeutic effect of 1,4-DPCA and DMOG, the inhibitors of P4H [38, 39], on the proliferation, invasion, migration, and apoptosis of PTC cells. First, the BCPAP and TPC-1 cells were treated with 1,4-DPCA or DMOG and a significant inhibition of their proliferation was observed compared to the control group (Fig. 9A, B). This outcome suggests that 1,4-DPCA and DMOG can effectively inhibit PTC cell growth. Additionally, the results of Transwell assays showed that treatment with 1,4-DPCA or DMOG significantly reduced the rate of migration of PTC cells compared to the cells of the control group (Fig. 9C, D). Likewise, the invasive ability of PTC cells was also markedly decreased after treatment with 1,4-DPCA or DMOG (Fig. 9E, F). These findings indicate that 1,4-DPCA and DMOG could effectively inhibit the invasive and migratory ability of PTC cells, thus preventing the spread of cancer cells. Finally, the effect of 1,4-DPCA or DMOG on the apoptosis of PTC cells was assessed. The treatment with 1,4-DPCA or DMOG significantly increased the rate of apoptosis in PTC cells compared to the effect on the untreated control group (Fig. 9G, H). Thus, 1,4-DPCA and DMOG could induce apoptosis in PTC cells, leading to cancer cell death. The data strongly prove that 1,4-DPCA and DMOG, the specific inhibitors of P4H, could effectively inhibit the proliferation, invasion, migration, and promote apoptosis of PTC cells, and that these may be promising candidates for developing new therapies for PTC.

## Discussion

In the current study, we used a multi-step process involving bioinformatics mining and molecular biology experiments and demonstrated the tumorigenic role of P4HA2 in the progression of PTC. Mechanistically, P4HA2 acts as a substrate for TRIM21-mediated ubiquitination to facilitate PTC progression via activating the glycolytic pathway. Inhibition of P4H suppresses the malignant behavior of PTC cells. Thus, our findings reveal a novel function and mechanism of P4HA2 in promoting the progression of PTC.

P4HA2 is an important member of the P4H family, playing a pivotal role in collagen synthesis through the unique composition of two identical alpha subunits and two beta subunits [40, 41]. Research on P4HA2 has mainly concentrated in the fields of bladder cancer [10], prostate cancer [14], melanoma [42] and other diseases. Therefore, our study fills the lacuna in research on the role of P4HA2 in PTC. Previous studies have reported that, P4HA2-mediated stabilization of HIF-1α promotes FGFR3 expression and increases erdafitinib resistance in bladder cancer [10]. Furthermore, P4HA1 can promote resistance to chemotherapy by regulating the HIF-1α-dependent cancer cell stemness [38]. However, we did not observe the interaction between P4HA2 and HIF-1α in PTC cells, nor did we observe any effect of P4HA2 on the expression of HIF-1α. This discrepancy may be attributed to the differences in tumor types studied compared to the above studies. Significantly, we identified another interacting molecule of P4HA2, namely TRIM21, through MS analysis. TRIM21 is an E3 ubiquitin ligase that carries out its functions through its RING domain [35, 36], which can promote the ubiquitination of its substrates through K48, K63, and other linkages, and accelerate the ubiquitination-mediated degradation of the substrate [36, 37, 43]. For instance, in nasopharyngeal carcinoma, TRIM21-mediated K48-linked ubiquitination promotes the degradation of LHPP and then downregulates TYK2-STAT1phosphorylation to inhibit the progression of nasopharyngeal carcinoma [37]. In colorectal cancer, TRIM21-mediated K63 ubiquitination reduces the stability of the hnRNPA2B1 protein, leading to a decline in nuclear export and translation of KRAS mRNA, and reduced activation of the MAPK signaling pathway, ultimately inhibiting the malignant progression of colorectal cancer [43]. The inhibitory role of TRIM21 is also observed in the progression of other cancers, including gallbladder cancer [44], breast cancer [45] and multiple mylenoma [46]. Consistent with the above research, our results also indicate that TRIM21 can inhibit the malignant function of PTC cells and ultimately exert anti-cancer effects in PTC. In this study, we found that TRIM21 can promote the K48 and K63-linked ubiquitination of P4HA2 through the RING domain, thereby reducing its stability, consistent with the function of TRIM21 itself (Fig. 9I).

According to the Warburg effect, cancer cells characterize an increased rate of glycolysis to meet the energy required for their abnormal proliferation and metastasis [47]. In the process of the metabolism of glucose into pyruvate and lactate, key enzymes such as glucose transporter 1 (GLUT1), hexokinases 2 (HK2), phosphoglycerate kinase 1 (PGK1), enolase 1 (ENO1) and lactate dehydrogenase A (LDHA) play a positive role in promoting this reaction [48–50, 22]. Our study demonstrated that after knocking down P4HA2, there was a decrease in mRNA and protein levels of the above-mentioned key enzymes in glycolysis, indicating that P4HA2 influences the energy metabolism in PTC cells via the glycolysis pathway (Fig. 9I). Coincidentally, a recent study also reported that P4HA2 promotes the growth, migration, and invasion of PTC cells through the glycolytic pathway, which is consistent with our results [51]. In addition, the study also suggests that P4HA2 is influenced by YTH domain family 3 (YTHDF3) and further promotes the malignant function of PTC cells through the Hippo signaling pathway [51]. Innovatively, our study highlights the role of P4HA2 in promoting multi-organ metastasis of PTC cells and that the expression of P4HA2 is regulated by ubiquitination mediated by TRIM21.

1,4-DPCA and DMOG are two P4H inhibitors that are currently used in research on the treatment of breast cancer [11, 38], colorectal cancer [52] and other diseases [53]. 1,4-DPCA can attenuate breast cancer cell proliferation and invasion by reducing the deposition of type I and IV collagen [11]. DMOG inhibits the metastasis of colon cancer cells through the occludin-p38 pathway [54]. DMOG can also reduce the occurrence of nasopharyngeal cancer by down-regulating p53 expression [53]. In our study, we found that 1,4-DPCA and DMOG could attenuate the malignant roles of PTC cells, consistent with their role in other tumors. Our study also indicates that P4HA2 can be used as a potential target for treating PTC. This speculation still needs to be verified through further investigations.

## Conclusion

In summary, our findings indicate that P4HA2 is a key factor in the progression of PTC, and that it plays a crucial role in the regulation of the malignant biology of PTC, as evidenced by the significant inhibition of these processes following P4HA2 knockdown. Transcriptomic and proteomic analyses after interfering with P4HA2 expression showed that P4HA2 mainly regulates the glycolytic pathway in PTC, and this was further confirmed by ECAR assay. We also identified TRIM21, located upstream of P4HA2 in the regulatory pathway, as an interacting protein of P4HA2. Specifically, TRIM21 promotes the K48-linked and K63-linked ubiquitination modification of P4HA2 through the RING domain and then mediates P4HA2 degradation through the proteasome. We found that the inhibitors of P4H can weaken the function of PTC cells, and P4HA2 expression is closely related to tumor size in patients with PTC, which indicates that P4HA2 is very likely a target for the treatment of PTC. These findings offer new insights into the molecular mechanisms of PTC and may lead to the development of potentially novel therapeutic strategies.

## Supplementary information

Supplementary Figure

Supplementary data 1

Supplementary data 2

Supplementary data 3

Supplementary data 4

uncropped original western blots

## Data Availability

The bioinformatics code involved in this study can be obtained from the corresponding author under reasonable requirements.
