# Supplementary material for: Prolyl 4-hydroxylase subunit alpha-2 acts as a TRIM21 ubiquitination substrate to promote papillary thyroid cancer progression via the glycolytic pathway
Source: Cell Death Dis. 2025 May 17;16(1):395. doi: 10.1038/s41419-025-07702-0 (PMC12084645; doi:10.1038/s41419-025-07702-0)
Supplement: Supplementary file 1 — Supplementary Figure [file 41419_2025_7702_MOESM1_ESM.docx]

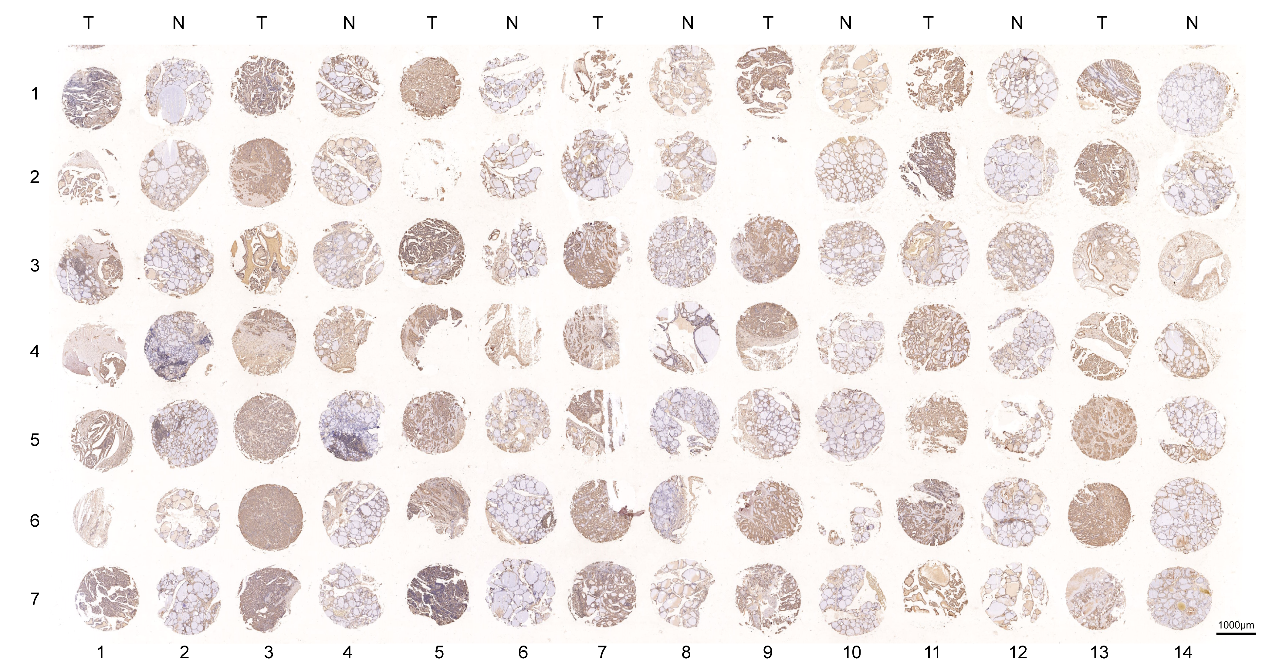


Supplementary Figure 1. Immunohistochemical (IHC) staining images of tissue microarray. The 1st, 3rd, 5th, 7th, 9th, 11th, and 13th columns in the figure are IHC staining images of PTC tissues, and the 2nd, 4th, 6th, 8th, 10th, 12th, and 14th columns are IHC staining images of the corresponding adjacent paracancerous tissues. Each row shows the IHC staining images of tumor tissues and adjacent paracancerous tissues of seven PTC patients. T represents tumor tissues and N represents adjacent paracancerous tissues. The scalebar represents 1000 μm.


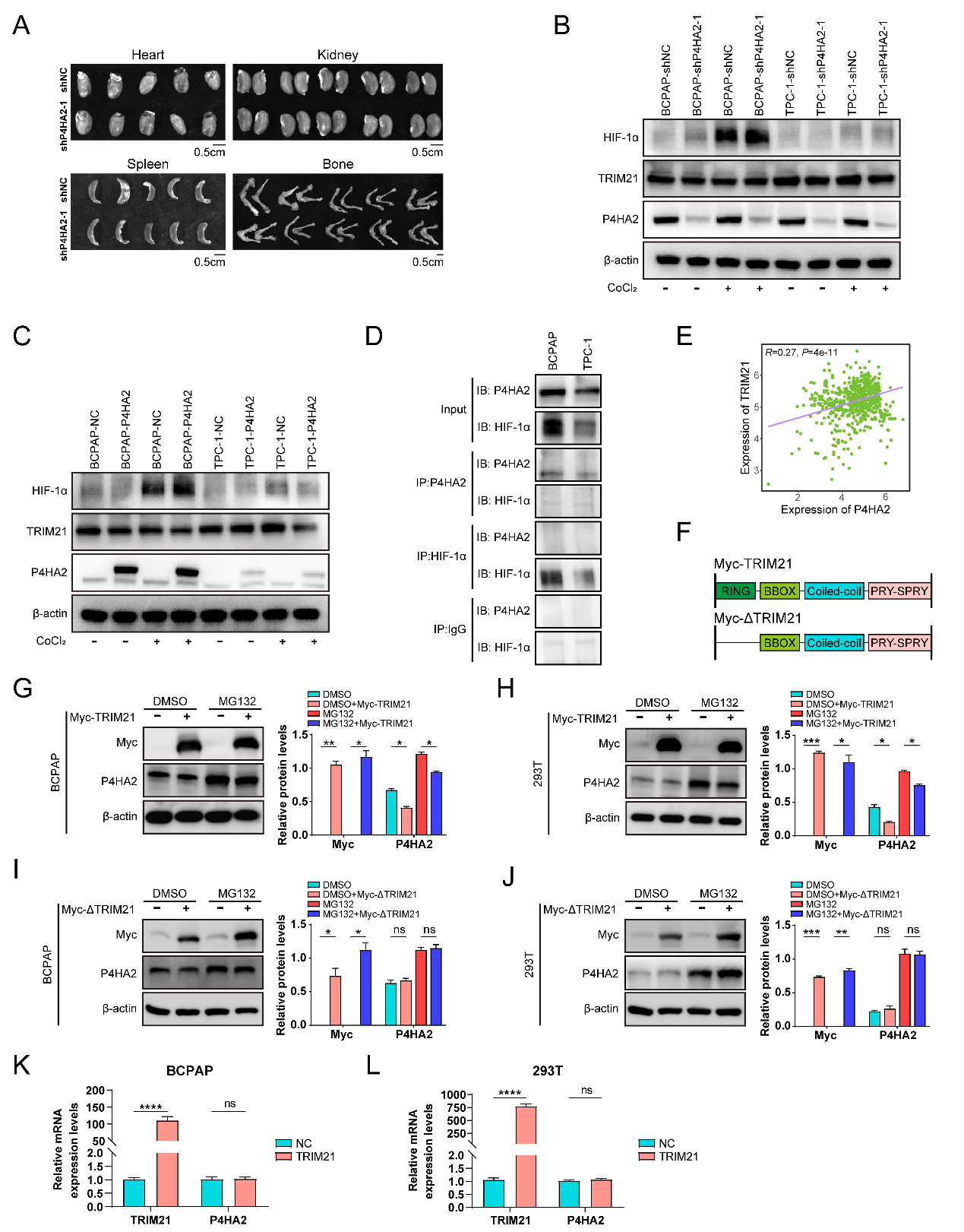


Supplementary Figure 2. A. Fluorescent images of mouse hearts, kidney, spleen and bones after injection of BCPAP-shNC cells and BCPAP-shP4HA2 cells in the tail vein. B-C. The expressions of HIF-1α and TRIM21 was detected in PTC cells by Western blotting assays. D. The interaction between P4HA2 and HIF-1α was detected through Co-IP. E. Correlation analysis between expression of P4HA2 and expression of TRIM21 in mRNA sequencing data of PTC patients from TCGA. F. Schematic diagram of TRIM21 and its truncated mutant lacking the RING domain (ΔTRIM21). G-J. BCPAP and 293T cells were transfected with Myc-TRIM21 (G-H) and Myc-ΔTRIM21 (I-J) plasmids for 48 h, then treated with MG132 or DMSO for 8 h, and the proteins were extracted for Western blotting. K-L. The expression of P4HA2 was detected in BCPAP and 293T cells overexpressing TRIM21 by RT-qPCR. * *P* < 0.05; ** *P* < 0.01; *** *P* < 0.001; **** *P* < 0.0001.
