# Supplementary material for: Prolyl 4-hydroxylase subunit alpha-2 acts as a TRIM21 ubiquitination substrate to promote papillary thyroid cancer progression via the glycolytic pathway
Source: Cell Death Dis. 2025 May 17;16(1):395. doi: 10.1038/s41419-025-07702-0 (PMC12084645; doi:10.1038/s41419-025-07702-0)
Supplement: Supplementary file 2 — Supplementary data 1 [file 41419_2025_7702_MOESM2_ESM.docx]

**Primer**

| Primer name | Primer forward sequences (5’-3’) | Primer reverse sequences (5’-3’) |
| --- | --- | --- |
| P4HA2 | GCTATGCTGTCTTCCAGTT | GCCATGGTGGTACCTACAGAA |
| TRIM21 | CAGAACTCAGGAGTGTGTGCCA | TCCAAGCCTCACTTGTCTCCGA |
| GAPDH | ATCACCATCTTCCAGGAGCGA | CCTTCTCCATGGTGGTGAAGAC |

**Sequence of shRNA**

| RNA name | Sequences (5’-3’) |
| --- | --- |
| shNC | TTCTCCGAACGTGTCACGTAA |
| shP4HA2-1 | CGAGATACTTTCAAGCATTTA |
| shP4HA2-2 | GCCGAATTCTTCACCTCTATTCTC |

**Sequence of siRNA**

| RNA name | Sequences (5’-3’) |
| --- | --- |
| siNC | UUCUCCGAACGUGUCACGU(dT)(dT) |
|  | ACGUGACACGUUCGGAGAA(dT)(dT) |
| siTRIM21-1 | GGUGAUAAUUGUCCUGGAA(dT)(dT) |
|  | UUCCAGGACAAUUAUCACC(dT)(dT) |
| siTRIM21-2 | CGCAGAGUUUGUGCAGCAA(dT)(dT) |
|  | UUGCUGCACAAACUCUGCG(dT)(dT) |

**Antibody**

| Primer name | Company | Category number | Application |
| --- | --- | --- | --- |
| P4HA2 | Thermo Fisher Scientific | PA5-118136 | Co-IP, WB, IHC, IF |
| TRIM21 | Proteintech | 67136-1-Ig | WB, IF |
| Flag | Zenbio | 390002 | Co-IP, WB |
| Myc | Proteintech | 16286-1-AP | Co-IP, WB |
| HA | Sigma | H6908 | WB |
| Anti-Ubiquitin (linkage-specific K48) | abcam | ab140601 | WB |
| Anti-Ubiquitin (linkage-specific K63) | abcam | ab179434 | WB |
| HIF-1α | abcam | ab51608 | Co-IP, WB |
| Rabbit IgG | Proteintech | 30000-0-AP | Co-IP, WB |
| Mouse IgG | Proteintech | B900620 | Co-IP, WB |
| HRP-conjugated IgG Fraction, Light Chain Specific | Proteintech | SA00001-7L | WB |
| Goat Anti-Rabbit IgG | Affinity Biosciences | S0001 | WB |
| Goat Anti-Mouse IgG | Affinity Biosciences | S0002 | WB |
| β-actin | abcam | ab8226 | WB |
