# Supplementary material for: Prolyl 4-hydroxylase subunit alpha-2 acts as a TRIM21 ubiquitination substrate to promote papillary thyroid cancer progression via the glycolytic pathway
Source: Cell Death Dis. 2025 May 17;16(1):395. doi: 10.1038/s41419-025-07702-0 (PMC12084645; doi:10.1038/s41419-025-07702-0)

Figrue 3A

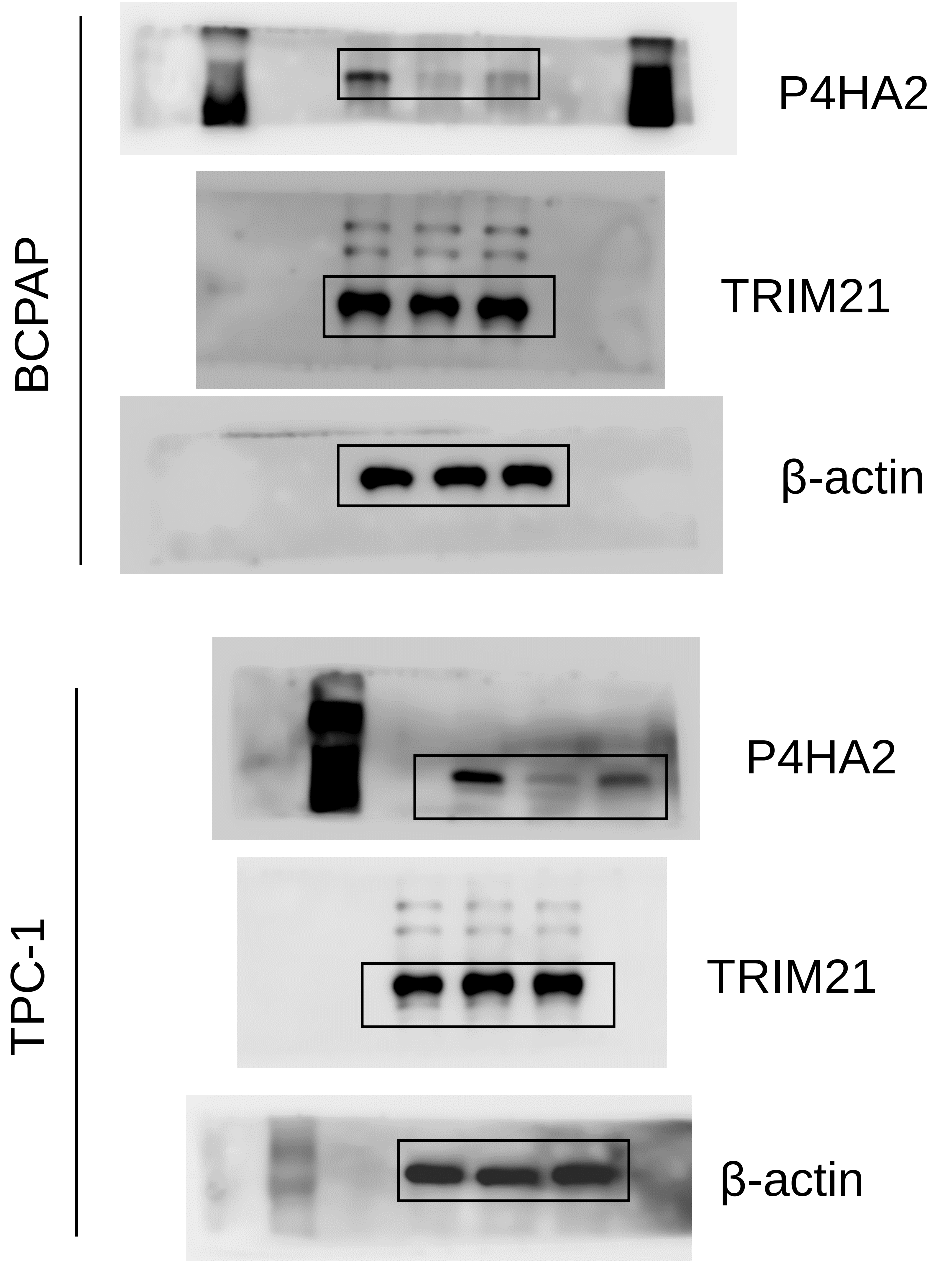

Figrue 4A

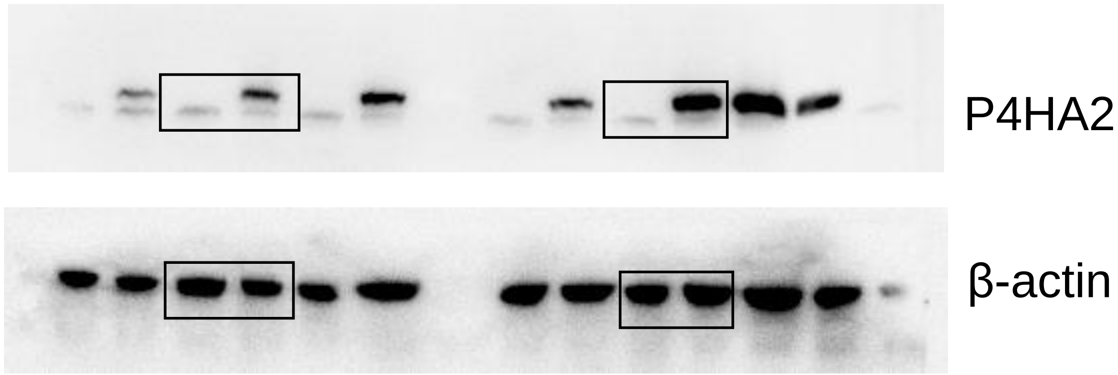

Figrue 7B

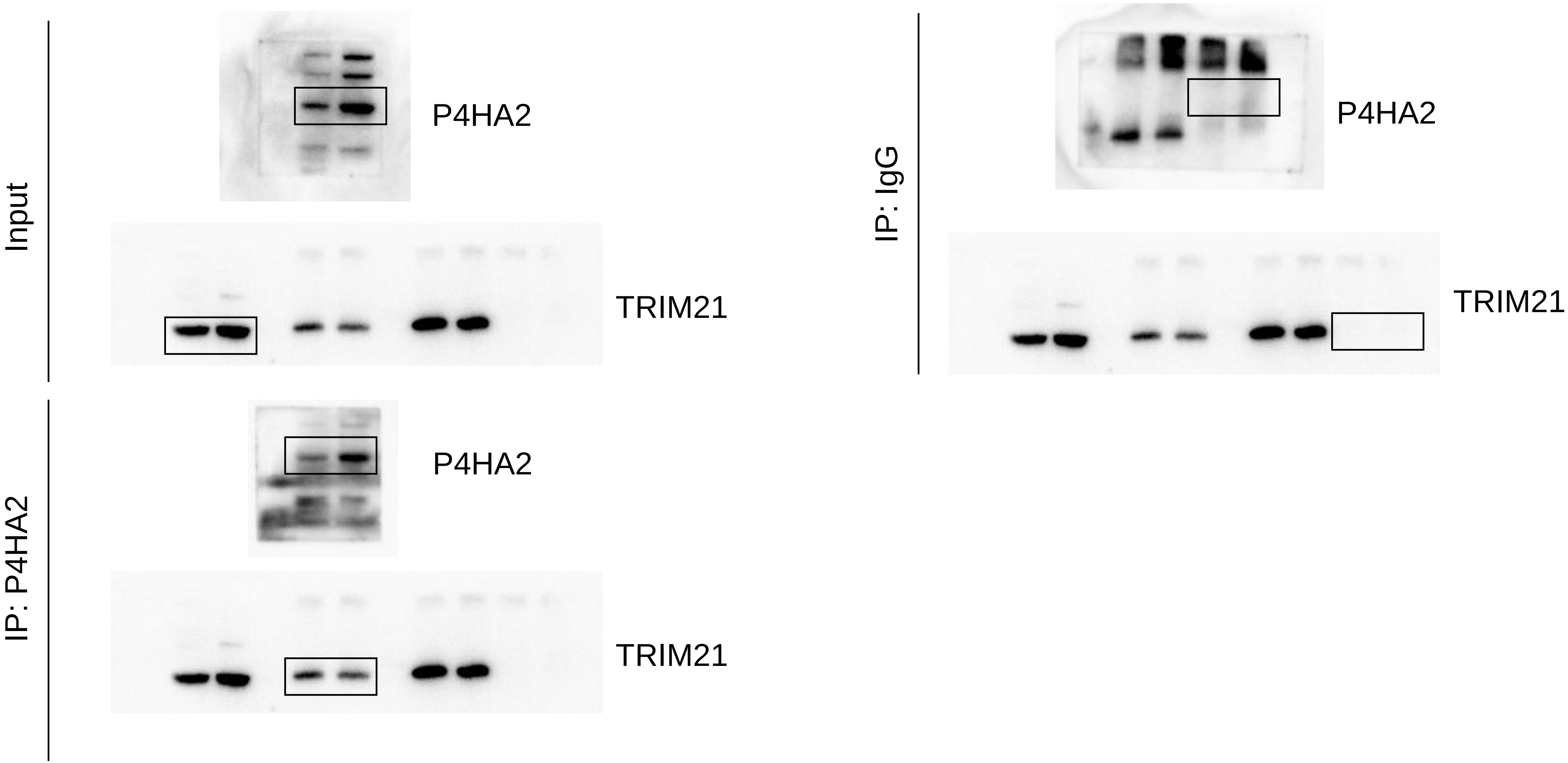
Input
P4HA2
TRIM21
IP: P4HA2
P4HA2
TRIM21
IP: IgG
P4HA2
TRIM21

Figrue 7D

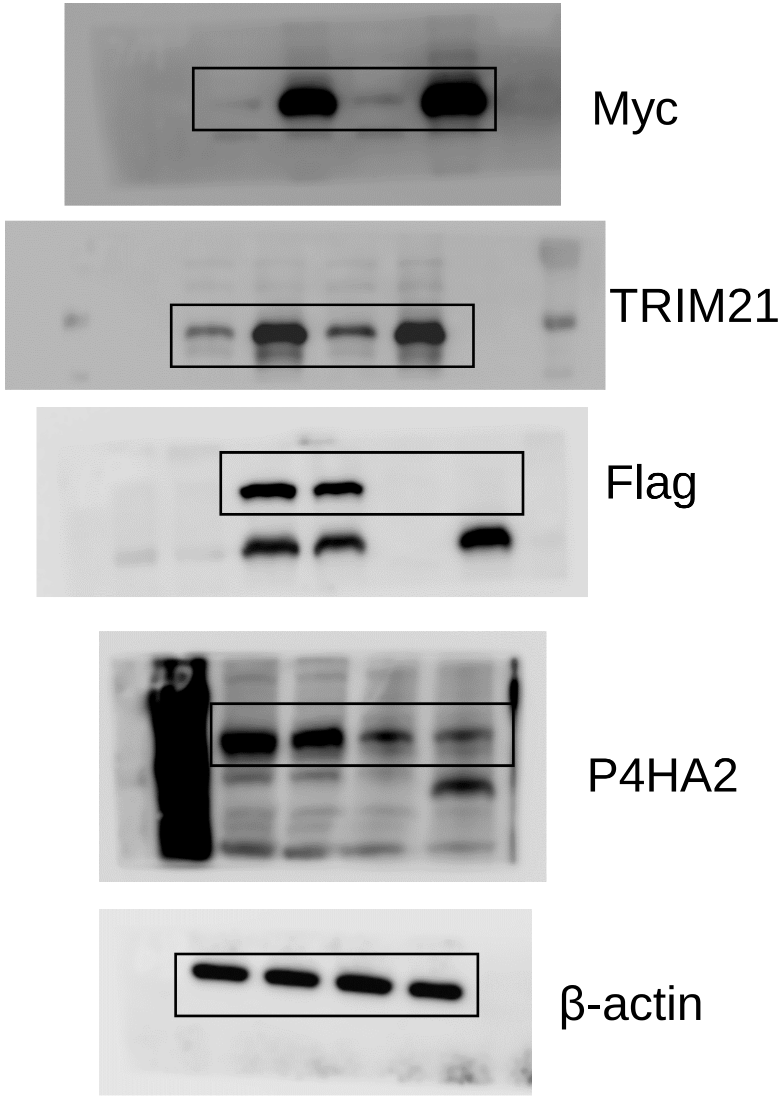

Figrue 7E

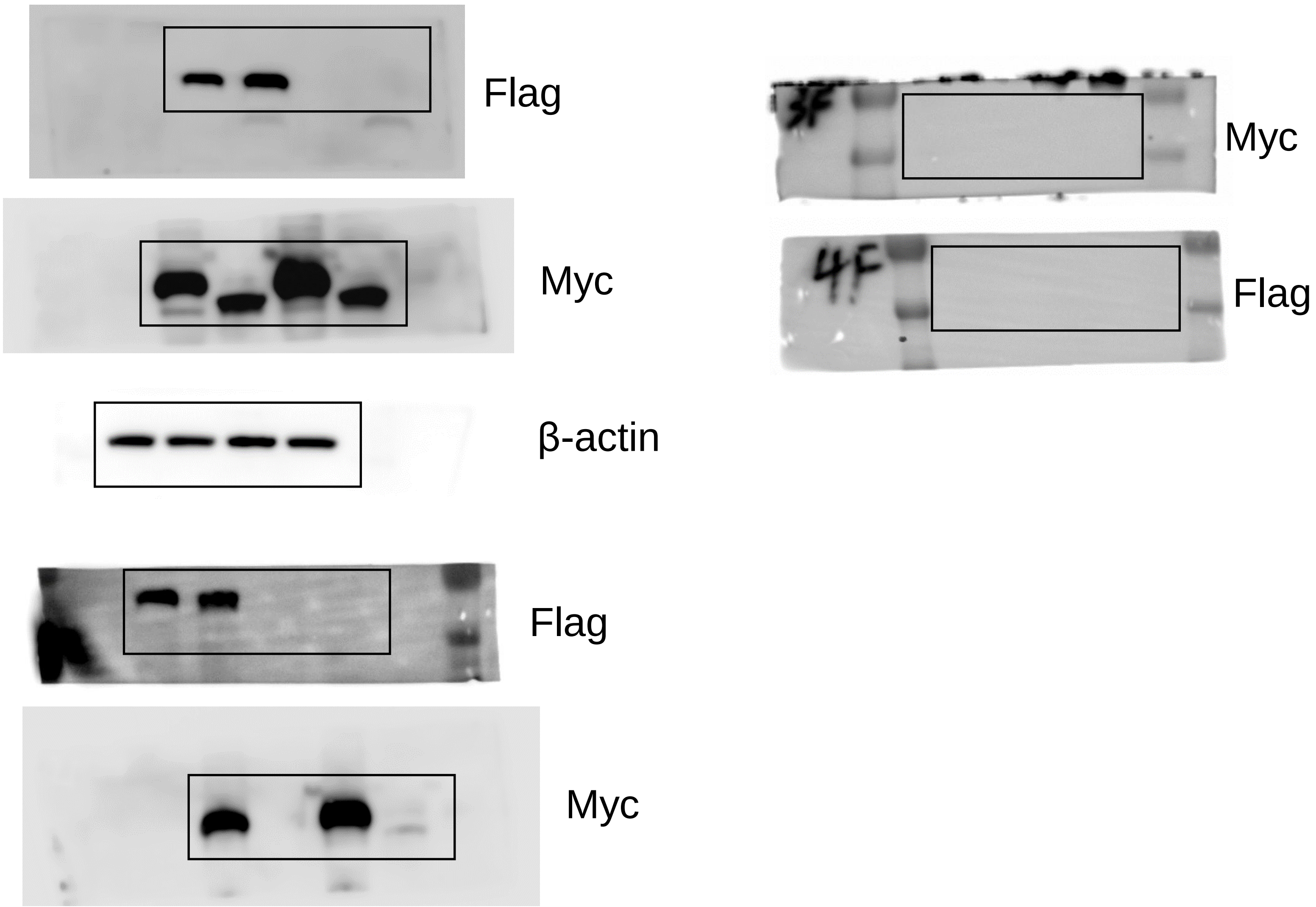

Figrue 7F

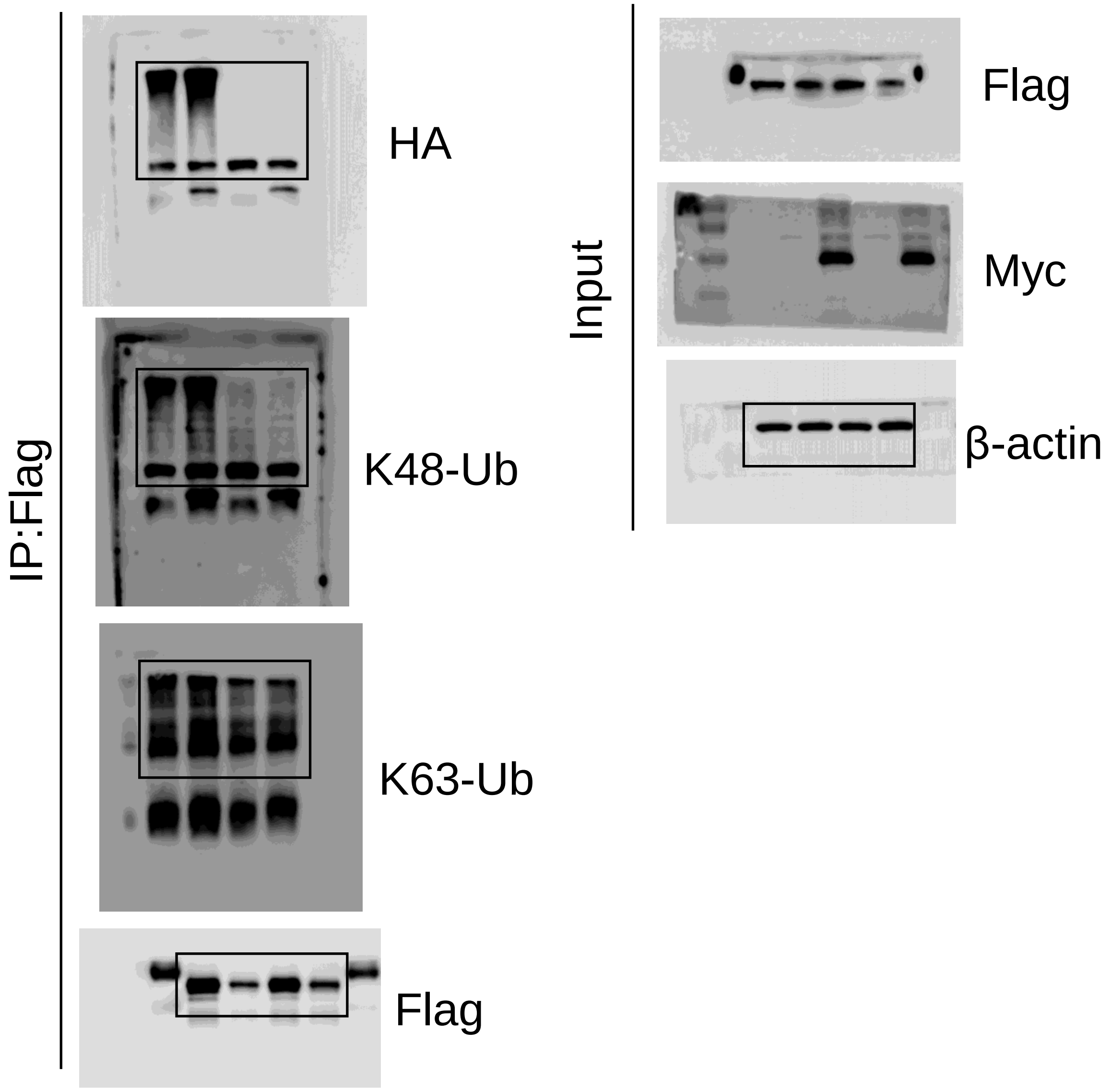

Figrue 7G

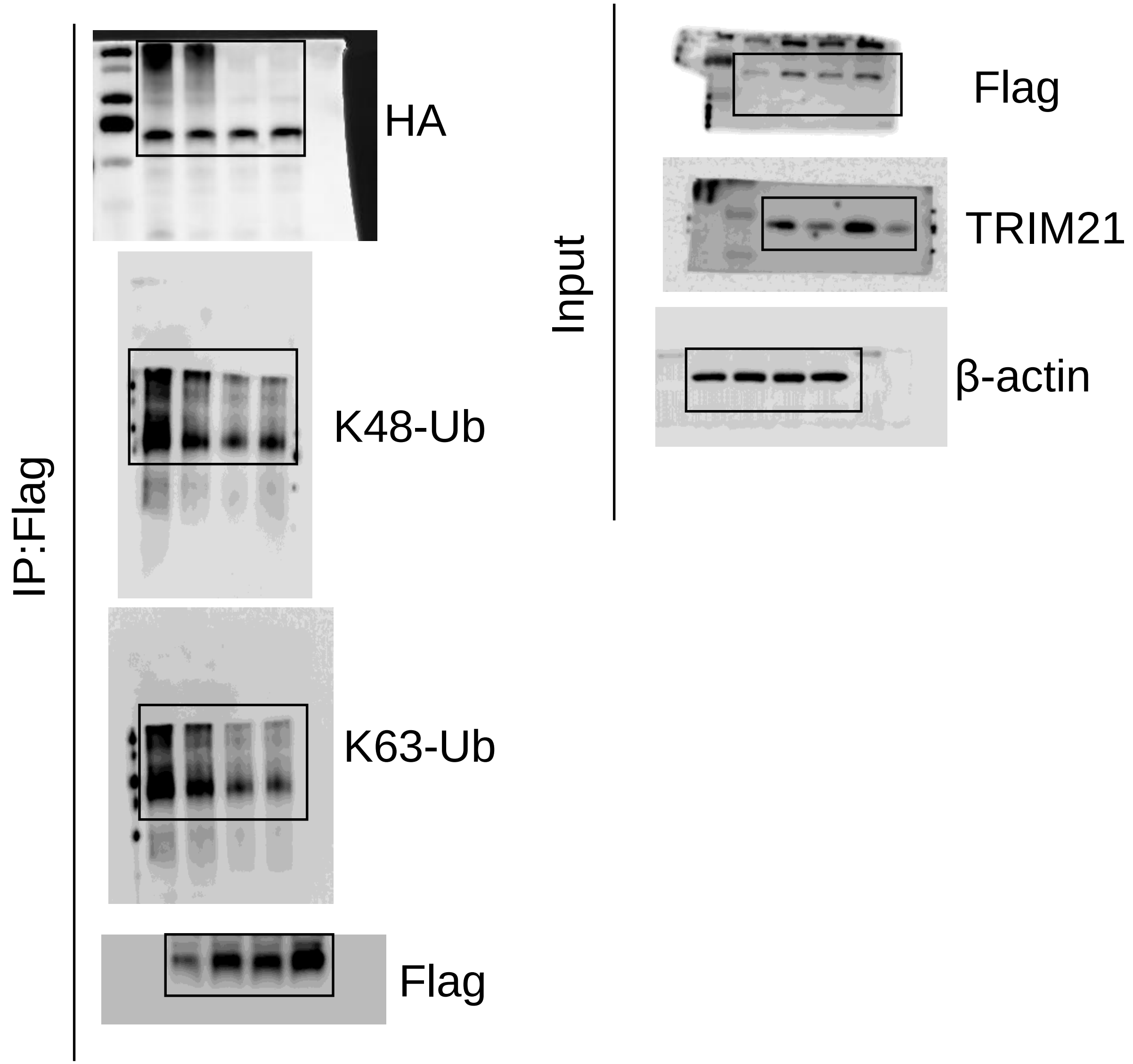

Figrue 7H

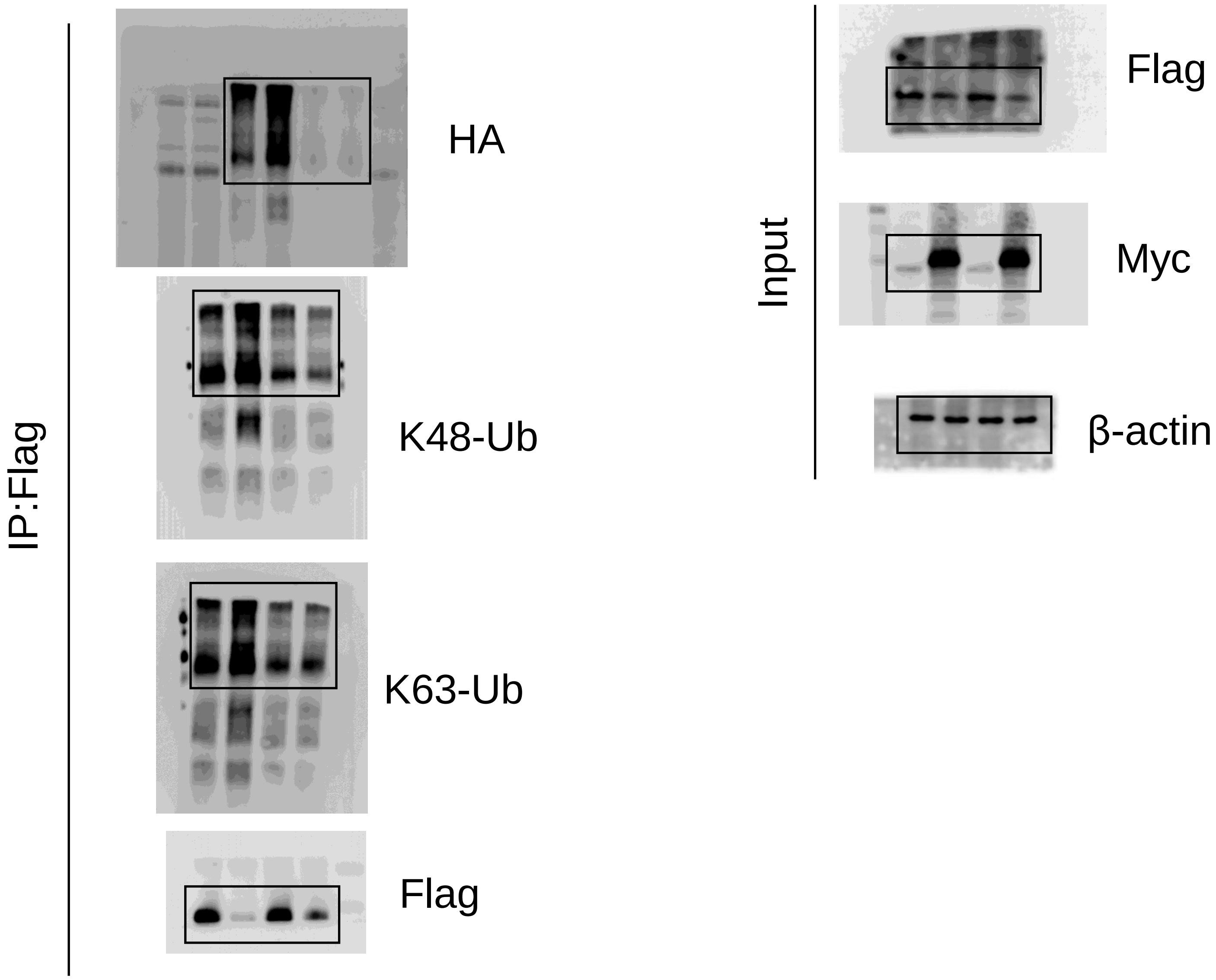

Figrue 7I

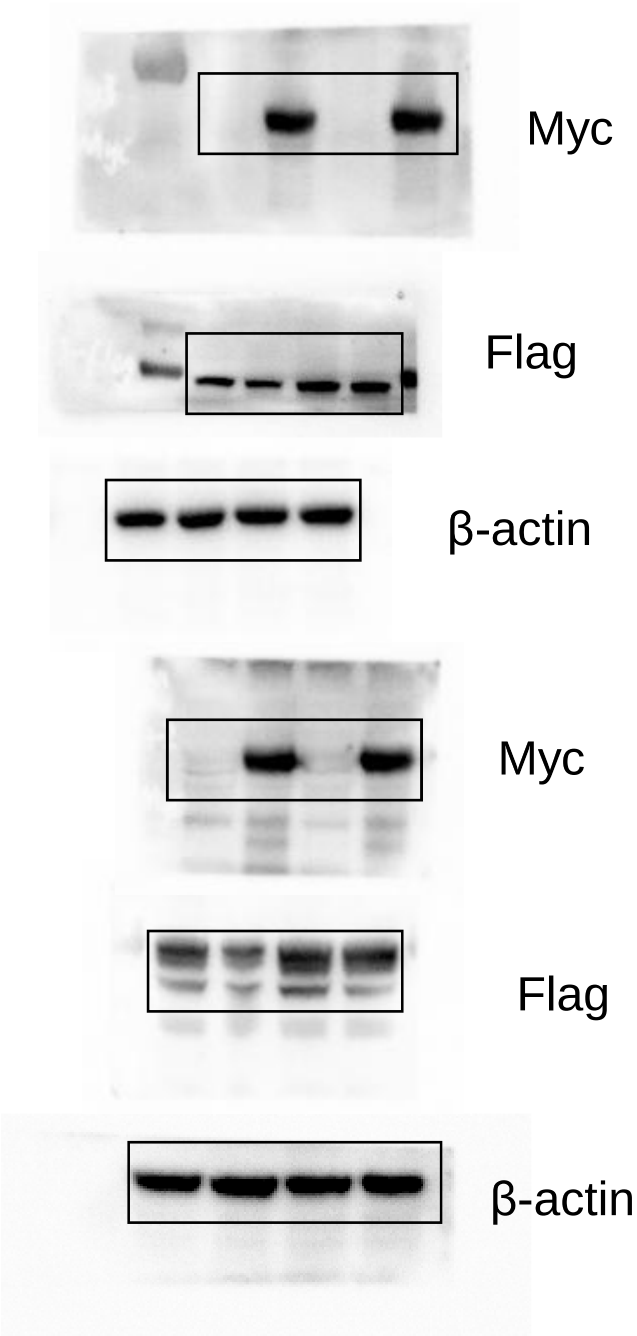

Figrue 7J

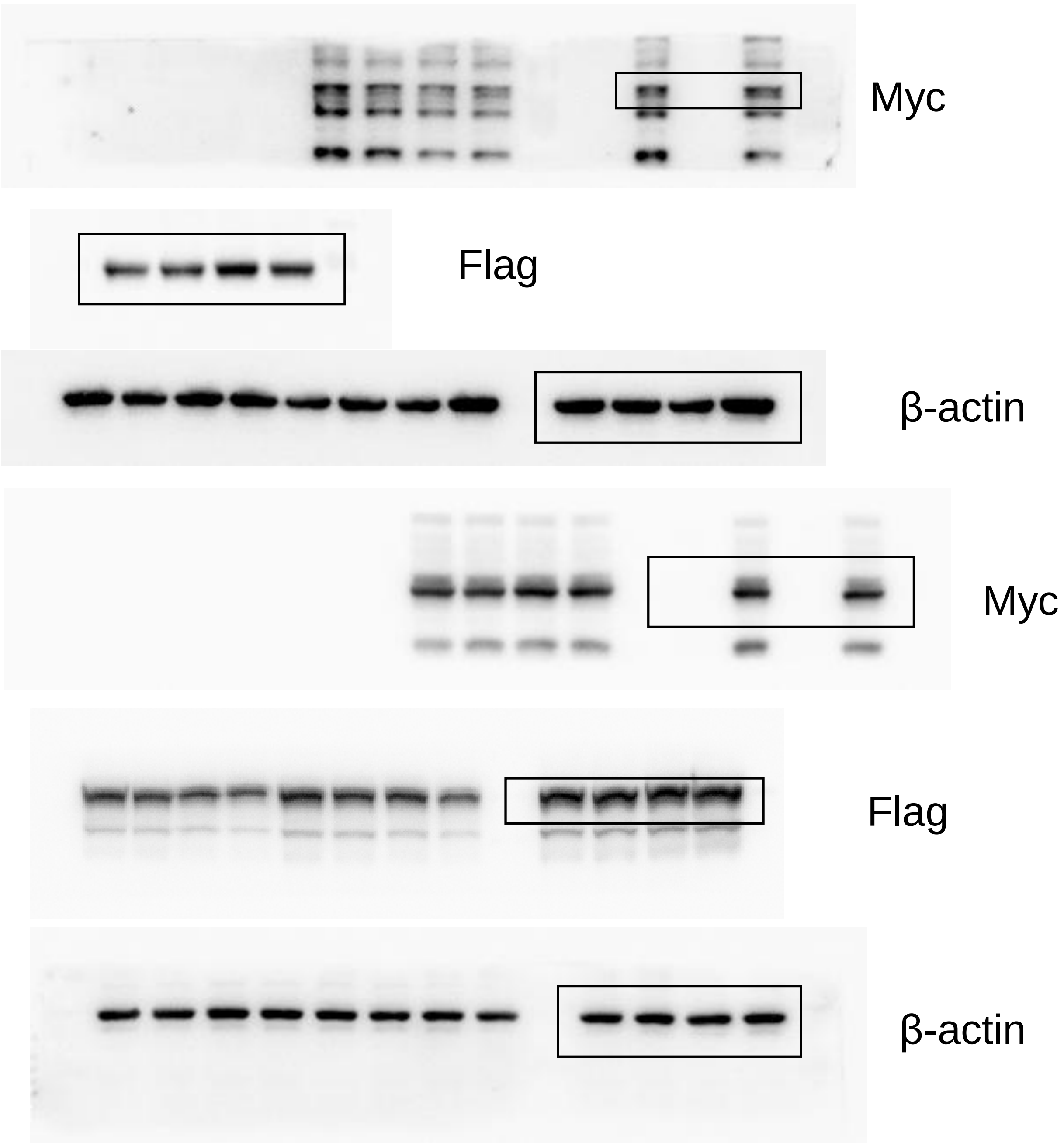

Figrue 7K

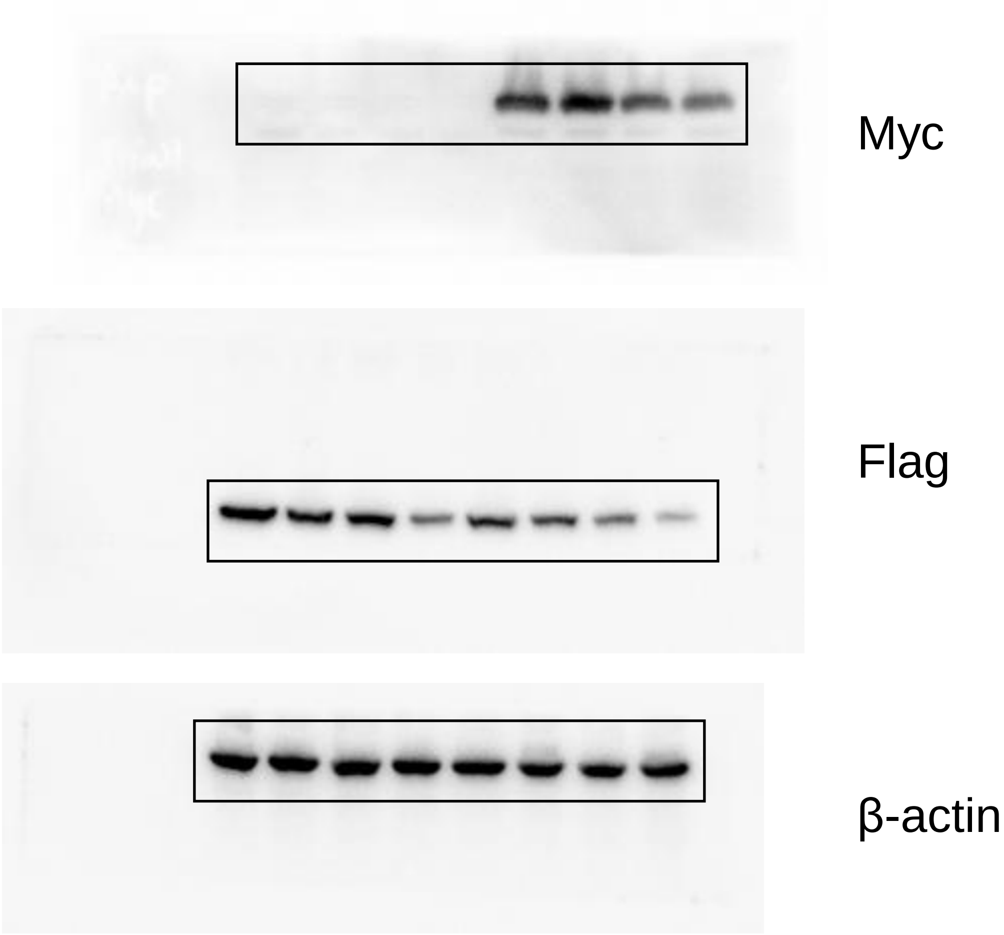

Figrue 7L

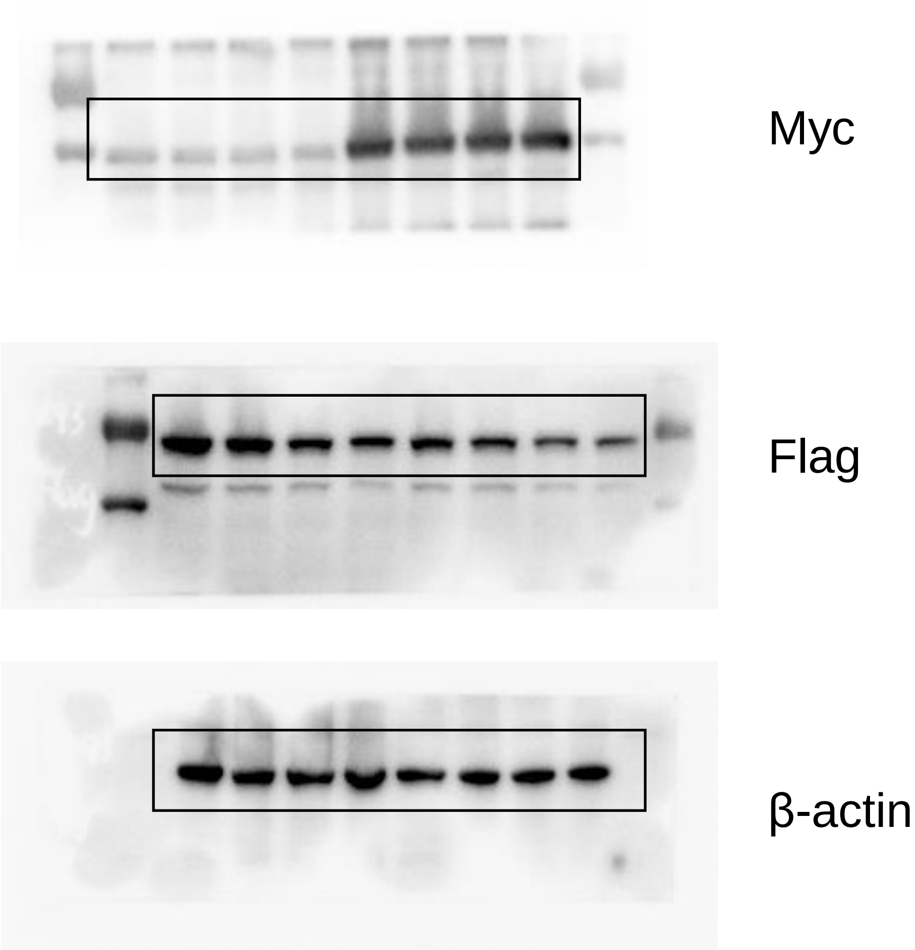

Figrue 7M

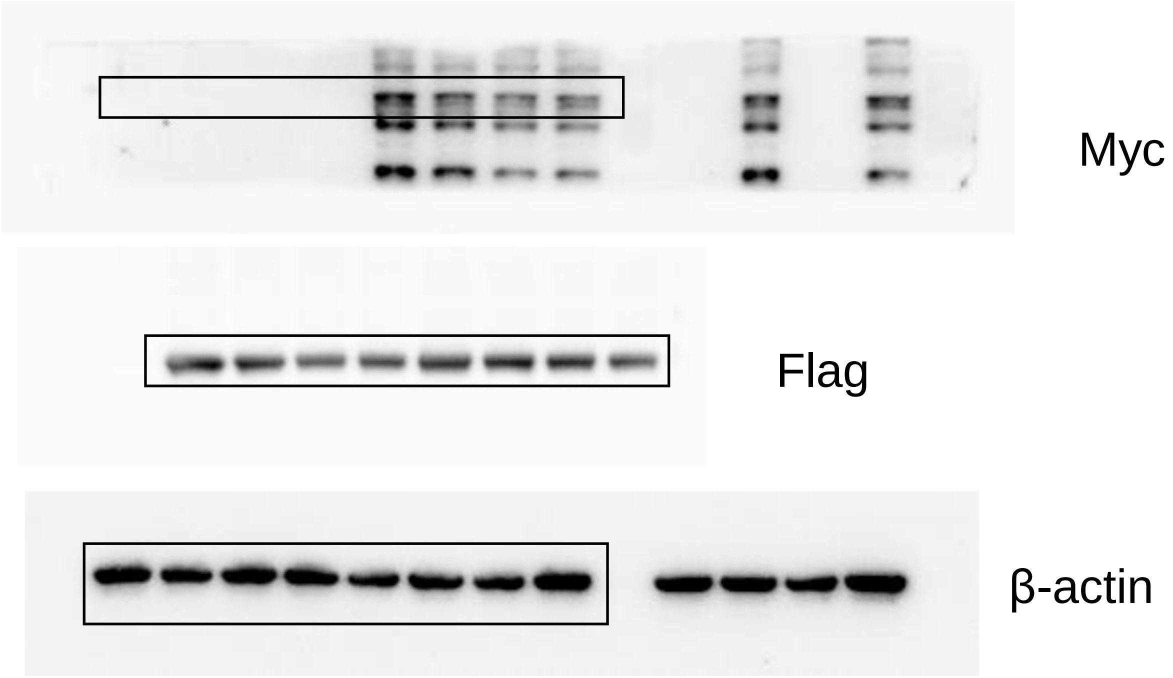

Figrue 7N

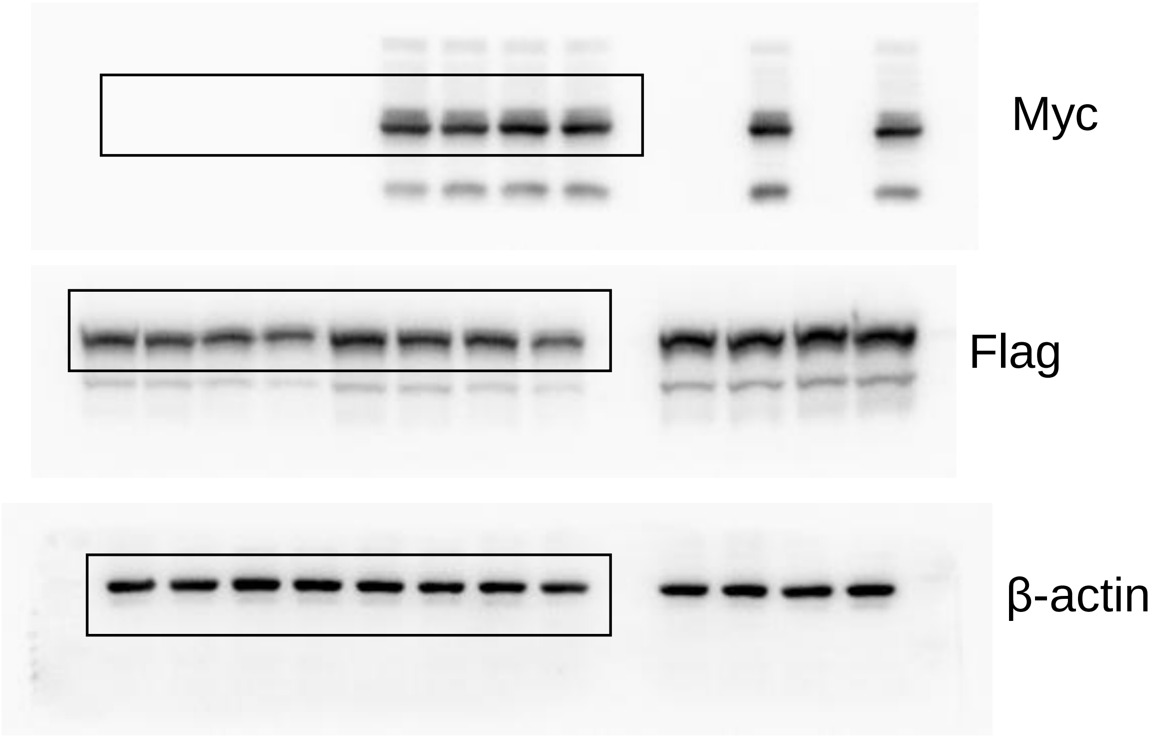

Figrue 8A

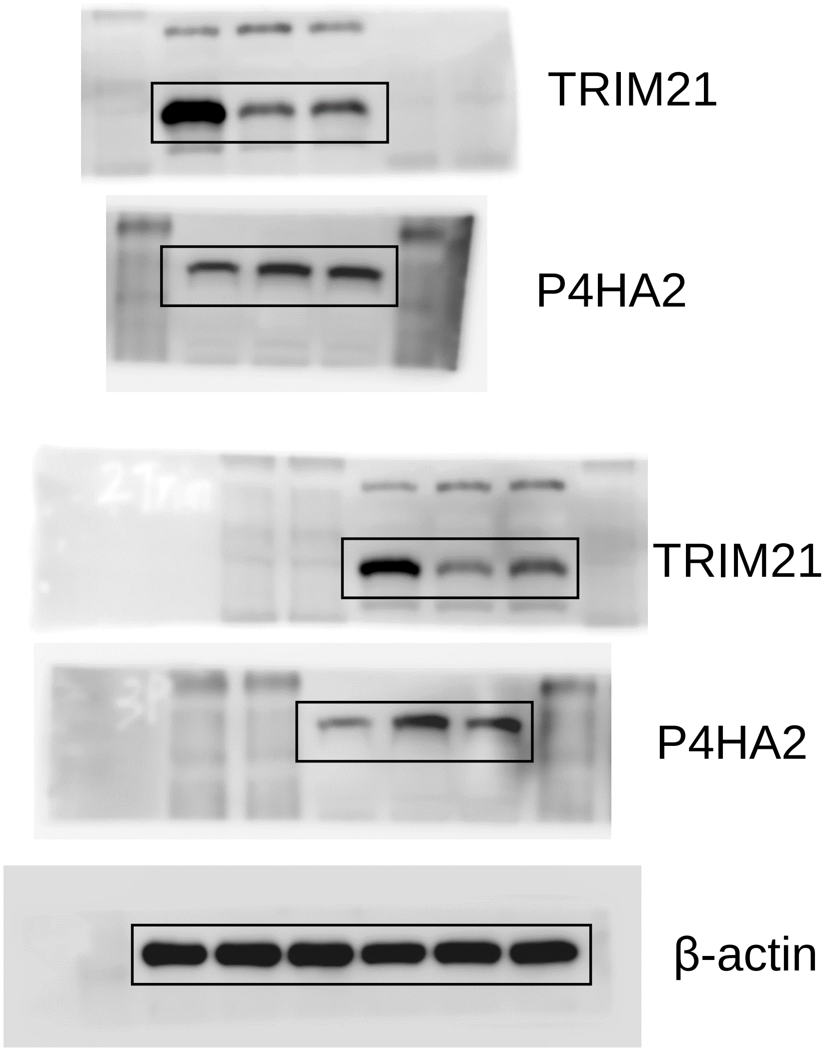

Supplementary Figure B

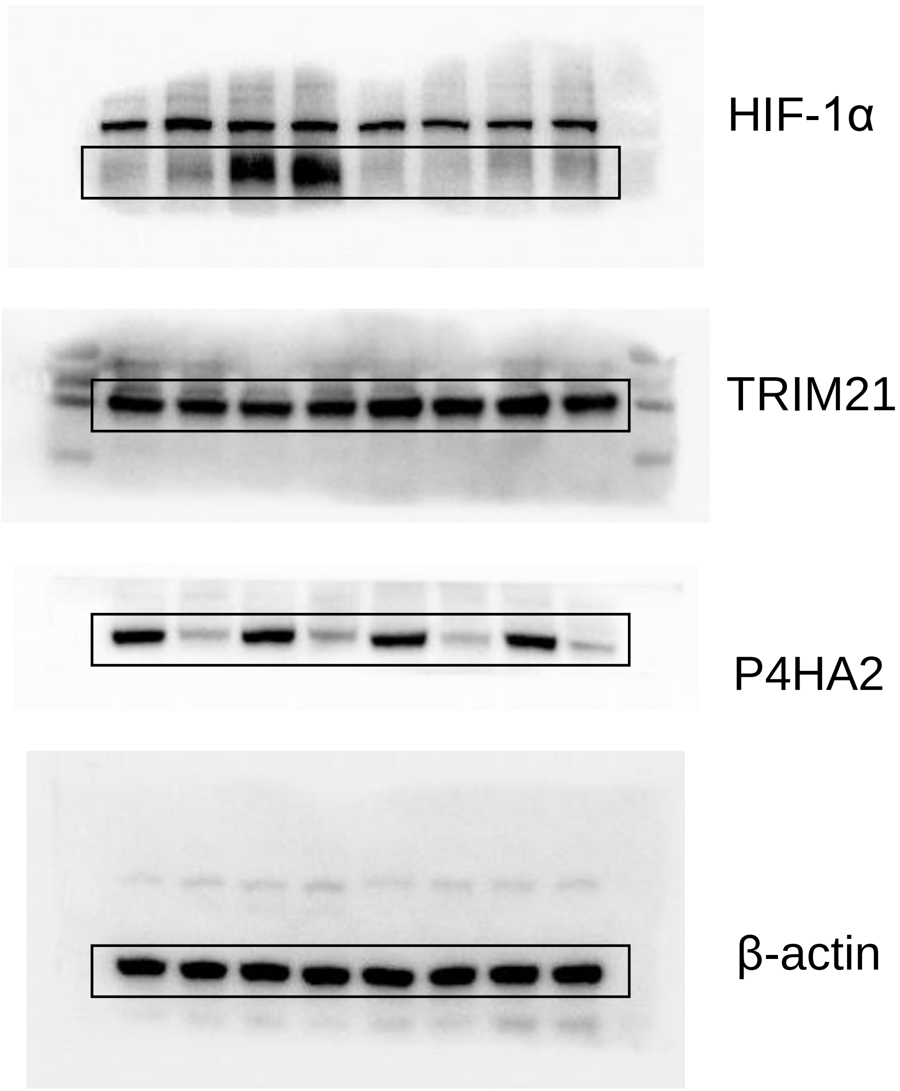

Supplementary Figure C

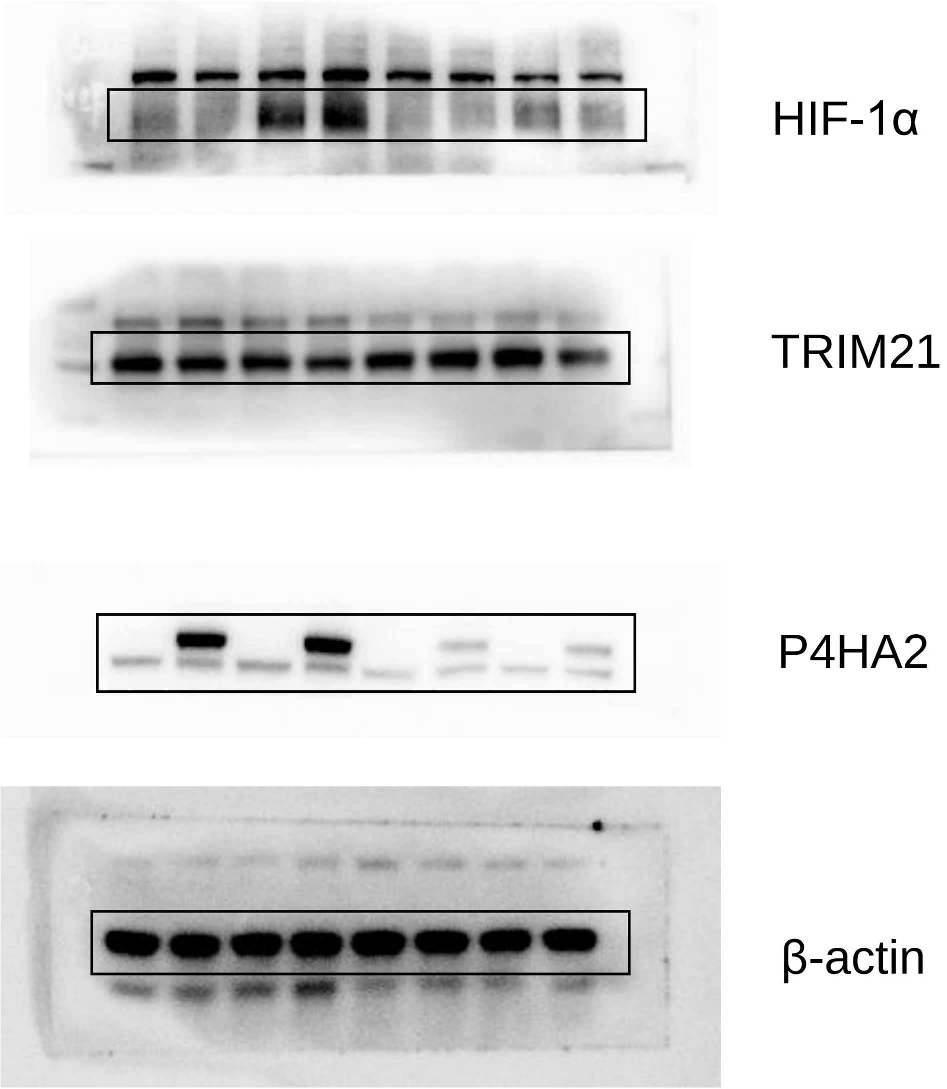

Supplementary Figure D

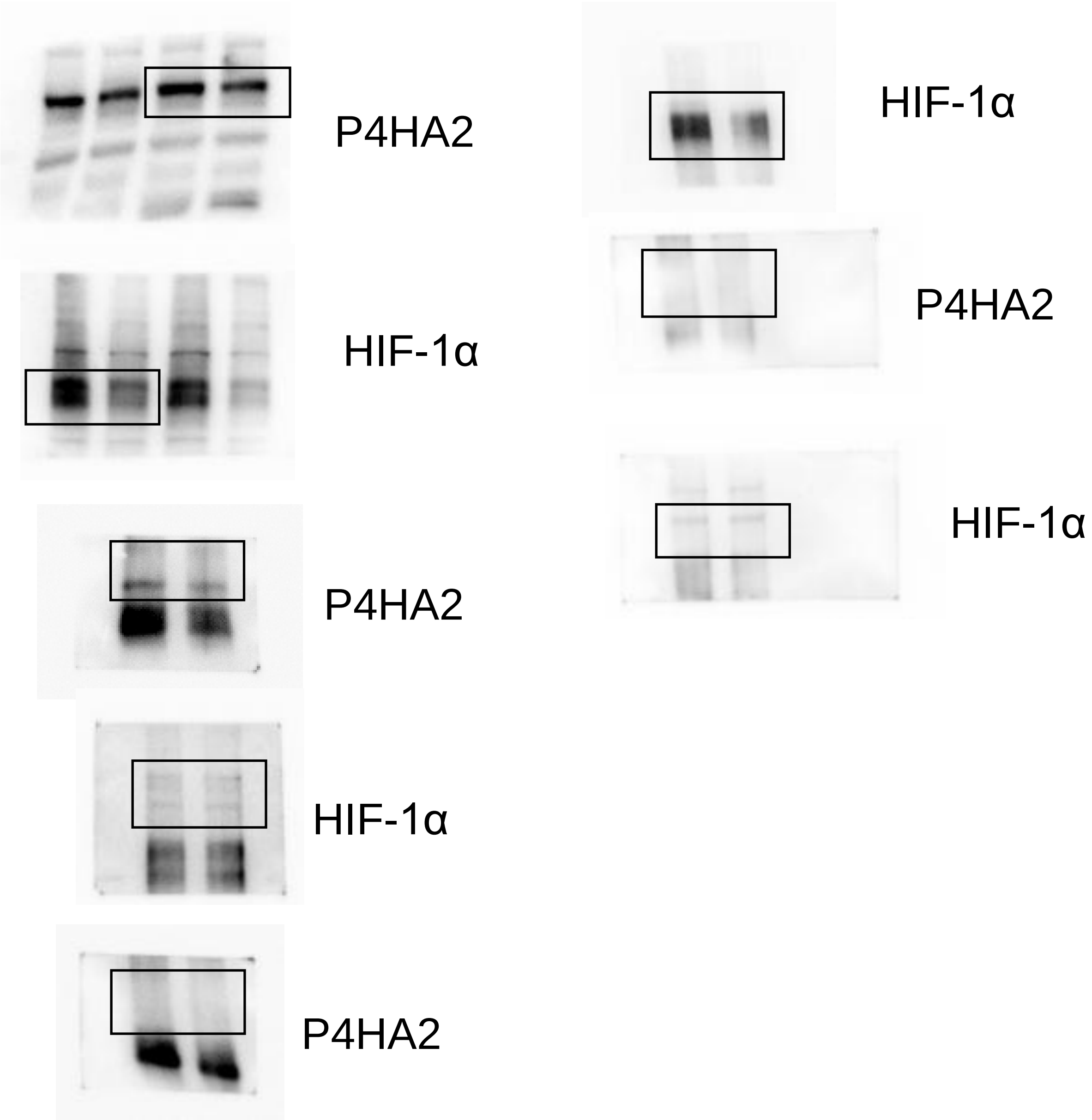

Supplementary Figure G

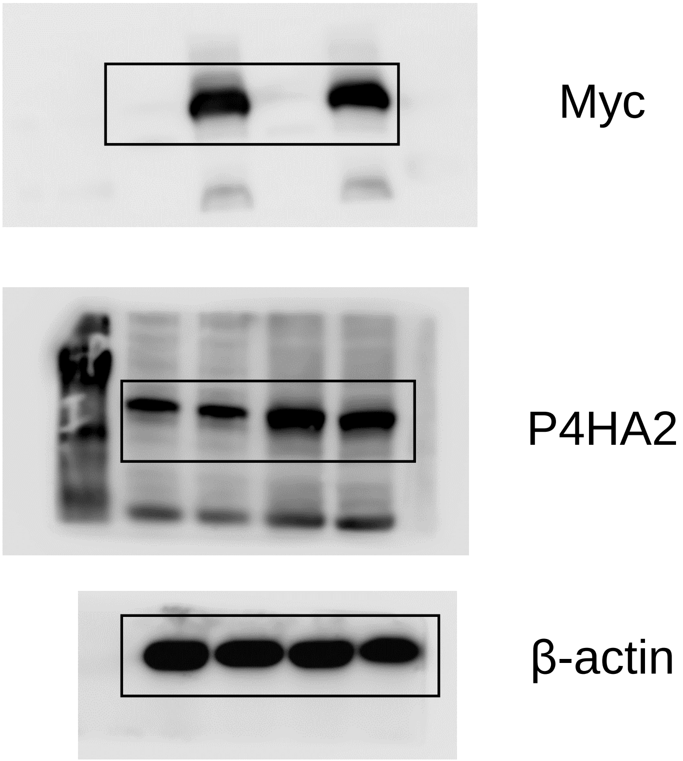

Supplementary Figure H

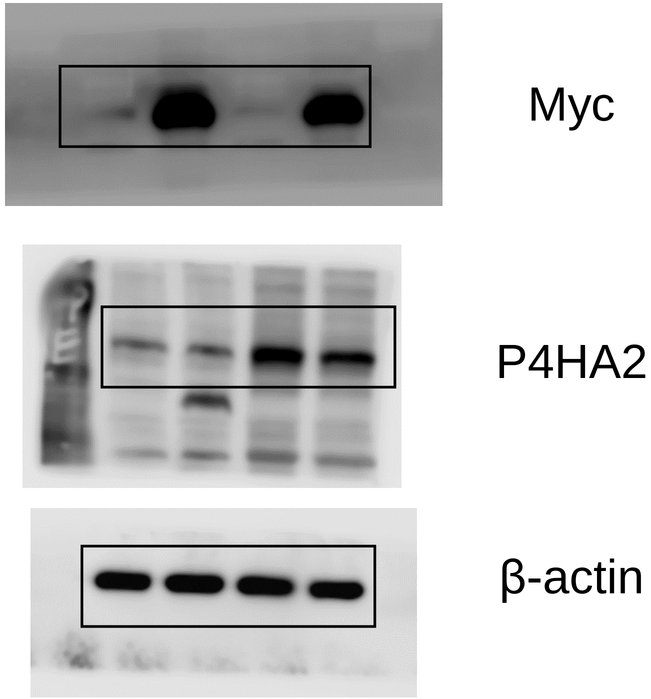

Supplementary Figure I

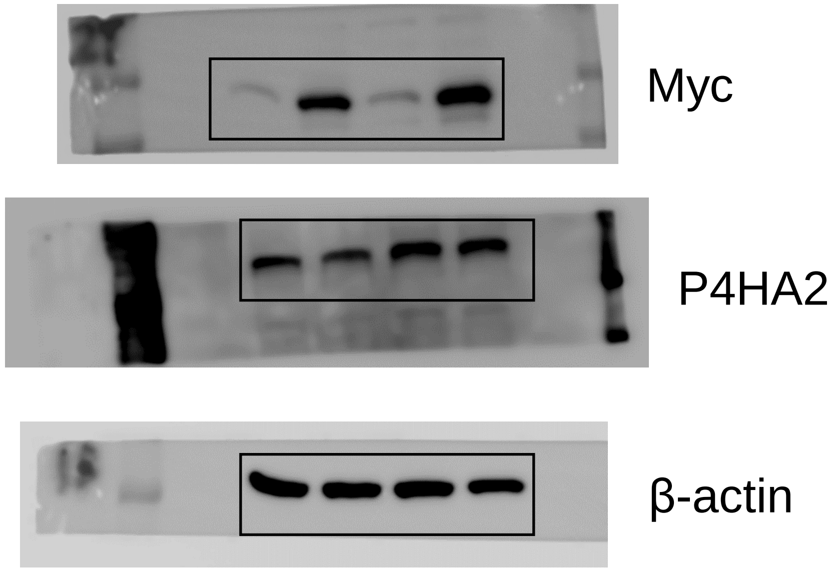

Supplementary Figure J

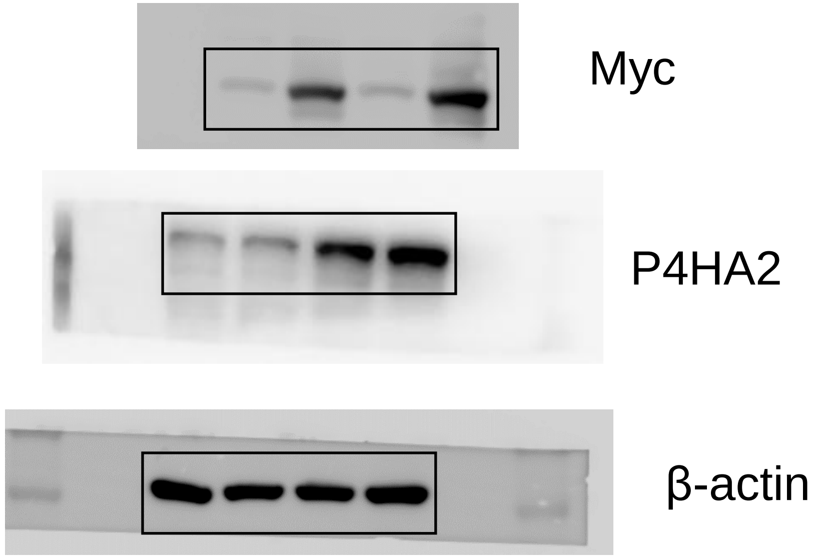

Supplement: Supplementary file 6 — uncropped original western blots [file 41419_2025_7702_MOESM6_ESM.pdf]
